# Effect of the Incorporation of Functionalized Cyclodextrins in the Liposomal Bilayer

**DOI:** 10.3390/molecules24071387

**Published:** 2019-04-09

**Authors:** Romina Zappacosta, Benedetta Cornelio, Serena Pilato, Gabriella Siani, François Estour, Massimiliano Aschi, Antonella Fontana

**Affiliations:** 1 Dipartimento di Farmacia, Università “G. d’Annunzio”, Via dei Vestini snc, I-66100 Chieti, Italy; r.zappacosta@unich.it (R.Z.); serena.pilato@unich.it (S.P.); gabriella.siani@unich.it (G.S.); 2 Normandie Univ, INSA Rouen, UNIROUEN, CNRS, COBRA (UMR 6014 & FR3038), 76000 Rouen, France; benedettacornelio@hotmail.com; 3 Dipartimento di Scienze Fisiche e Chimiche, Università di L’Aquila, Via Vetoio snc, I-67100 L’Aquila, Italy; massimiliano.aschi@univaq.it

**Keywords:** cyclodextrins, liposomes, nanocarriers, drug-delivery systems, molecular dynamics simulations, membrane stability

## Abstract

Liposomes loaded with drug–cyclodextrin complexes are widely used as drug delivery systems, especially for species with low aqueous solubility and stability. Investigation of the intimate interactions of macrocycles with liposomes are essential for formulation of efficient and stable drug-in-cyclodextrin-in-liposome carriers. In this work, we reported the preparation of unilamellar vesicles of 1-palmitoyl-2-oleoyl-*sn*-glycero-3-phosphocholine (POPC) embedded with native β-cyclodextrin and two synthetic derivatives: heptakis(2,3,6-tri-*O*-methyl)-β-cyclodextrin (TMCD) and heptakis(2,3-di-*O*-acetyl)-β-cyclodextrin (DACD). We then studied the effect of these macrocycles on the liposomal size, membrane viscosity, and liposomal stability at different temperatures and concentrations. We observed that TMCD and DACD affected vesicle size and the change of size was related to CD concentration. Irrespective of its nature, the macrocycle established interactions with the phospholipidic head groups, preventing cyclodextrins to diffuse into the lipid bilayer, as confirmed by molecular dynamics simulations. Such supramolecular structuring improves liposome stability making these colloid systems promising carriers for biologically active compounds.

## 1. Introduction

Nanocarriers are able to change the physicochemical properties of the incorporated molecules, affect the pharmacokinetic profiles of embedded drugs, as well as allow the incorporated molecules to overcome biological barriers. For these reasons nanovehicles are used to enhance the effectiveness of the drug, decrease severe side effects, protect the drug from chemical degradation that may compromise their efficiency [1], or lead to the formation of secondary products, which are sometimes toxic. 

Liposomes are spherical lipid vesicles formed in aqueous solution by phospholipid molecules organized in bilayers enclosing an aqueous compartment. These aggregates are useful in biological, biomedical, and biotechnological applications as drug delivery systems due to their extraordinary capacity to encapsulate hydrophilic drugs in the aqueous core and to trap lipophilic compounds within the membrane [2].

Nevertheless, the incorporation of highly hydrophobic molecules into liposomes can destabilize the lipid membrane, leading to a rapid release of the drug from the bilayer. In an attempt to overcome this problem, in 1994, McCormack and Gregoriadis engineered, for the first time, the possibility of forming a combined system of cyclodextrins (CDs) and liposomes called drug-in-cyclodextrin-in-liposomes (DCLs) [3]. These systems are able to combine the capability of CDs to form CD/drug inclusion complexes with the use of liposomes as shuttle systems. Recently, different DCLs have been designed to overcome drawbacks such as the low solubility and low stability of many type of active compounds comprising aromatics, essential oils, and hydrophobic drugs [4,5,6,7,8,9,10,11,12,13].

CDs are water soluble oligosaccharides that contain hydrophobic cavities in which they can accommodate via host–guest interactions hydrophobic drugs while their outer surface permits aqueous miscibility. The embedded drug molecule (guest molecule) is situated in the cavity of CD (host molecule) without significantly affecting the host framework structure. The native and most common CDs are composed of six (α-CD), seven (β-CD), and eight (γ-CD) glycosyl units [14]. In order to increase their aqueous solubility, different chemical modifications have been designed to improve water solubility of natural occurring CDs [15,16,17,18,19,20].

DCLs combine the advantages of both carriers and thereby provide a controlled drug release [13,21,22,23]. The DCL system was applied to encapsulate a variety of lipophilic drugs [4,24,25].

Despite the increasing interest on DCLs, often including randomly substituted CD, the effects of differently functionalized single isomer CDs on the stability and fluidity of lipid bilayers are still poorly understood. The presence of CDs in the aqueous compartments of liposomes demonstrated to affect liposome characteristics and membrane integrity depending on CD functionalization and liposome composition and structure [26,27,28,29,30].

**Figure 1:**
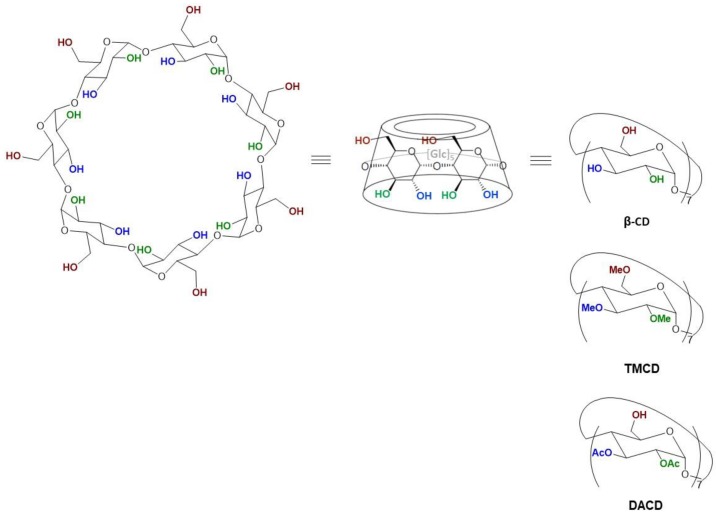
β-Cyclodextrins and functionalized cyclodextrins (CDs).

In order to deepen the mechanisms and capacity of functionalized CDs to interact with phospholipidic membranes, we investigated the effect of some entrapped modified CDs (Figure 1), at different phospholipidic ratio, in the inner aqueous phase of pure 100-nm 1-palmitoyl-2-oleoyl-*sn*-glycero-3-phosphocholine (POPC) unilamellar liposomes on membrane chemicophysical characteristics and correlated it with CDs affinity with the phospholipidic bilayer. In particular we selected three CDs having different properties, especially a distinct solubility in water and in an organic medium such as chloroform, the native (nonfunctionalized) β-CD was therefore chosen as a reference molecule. The heptakis(2,3,6-tri-*O*-methyl)-β-cyclodextrin (TMCD) [31,32] was used because it was demonstrated to interact with the lipid bilayer [33]. The last derivative—heptakis(2,3-di-*O*-acetyl)-β-cyclodextrin (DACD) [34,35]—to the best of our knowledge, had never been used for studying liposome interactions. Experimental evidence were also confirmed by molecular dynamics (MD) simulations, in particular, Umbrella Sampling (US) calculations, aimed at quantifying the energetics of cyclodextrin–membrane (mimicking the liposome) interaction. The comprehensive experimental and theoretical results allowed us to clarify the behavior of DLCs and tune and optimize the DCLs formulation.

## 2. Results and Discussion

### 2.1. Liposome Size

The liposome dispersions are quite homogenous (Table 1), with the polydispersity of each dispersion always lower than 0.3. On addition of CDs the size of liposomes slightly increases. This increase is then approximately correlated to the concentration of added CD. Since the ability of CD to break and solubilize liposomes has been demonstrated for concentrations from 100 to 1000 higher than those investigated in this study [27], the increase in dimensions is probably related to the capacity of CDs to interact with the liposomal surface without disrupting completely the vesicles. The effect is particularly evident for functionalized CDs. In the case of POPC/TMCD the increase could be partly related to the capacity of TMCD, as a consequence of the presence of three additional methyl groups per glycosidic unit, to interact more deeply with the bilayer partly contacting the methylene groups of the alkyl chains close to the head moieties, in agreement with previous measurements [33,36]. Due to its high water solubility, no evidence of TMCD embedding in the bilayer was obtained from viscosity and stability measurements (see below). It is likely that TMCD entails additional hydrophobic interactions mostly at the level of the POPC head groups and these interactions affect the radius of the liposomes. It is worth mentioning that the specific interactions observed [36] for TMCD and 1,2-dipalmitoyl-*sn*-glycero-3-phosphocholine (DPPC), supposed to penetrate the hydrophobic region of the bilayer, could not hold for POPC. Indeed it has been demonstrated that different phospholipids interact differently with CDs, despite vesicles composed of saturated phospholipid were found to be mainly more stable than vesicles obtained with nonsaturated phospholipids [27].

In the case of DACD, the presence of two additional acetyl groups per glycosidic unit with respect to β-CD may affect the interaction with the liposomes. The effect may be connected to the capacity of ester groups to be a good hydrogen bond acceptor [37]. Recent studies have highlighted that surfactants containing specific moieties such as amide bonds are able to stabilize POPC liposomes thanks to the capability of these surfactants to form strong hydrogen bonds (HB) with the neighboring phospholipids of the membrane [38,39]. Likely DACD is able to organize, through additional hydrogen bonds, water molecules on the phospholipidic surface thus accounting for the increase of liposomal dimensions (i.e., DLS monitors the hydrodynamic diameter and therefore takes into account the solvated liposomes).

### 2.2. Viscosity of the Membrane

Measurement of membrane viscosity allows to get information on the organization and packing of POPC in the bilayer following the interaction with proper guests. The microviscosity is defined as the homogenous solution viscosity, which results in the same behavior as that in the microenvironment of the phospholipidic bilayer [40]. Therefore, we measured the microviscosity of the lipid bilayer of liposomal membranes (Table 2) by using the capacity of a fluorescent probe, i.e., pyrene, to form excimer when solubilized in a proper lipophilic environment [41]. The excimer forms from the encounter of an electronically excited pyrene molecule and another pyrene molecule in its ground state [42]. The ratio of the fluorescence intensities associated to the excimer and that of the monomer (I_E_/I_M_) provides details on the translational diffusivity of the probe pyrene in the bilayer. The obtained data refer to the microviscosity of the system [41,43] since a low value of I_E_/I_M_, associated to a low mobility of the pyrene within the bilayer, implies a high viscosity of the system whereas the C′_h_ value, i.e., the reciprocal of the slope of the straight line obtained by plotting I_E_/I_M_ vs. pyrene concentration (see Experimental Section 4.4), is proportional to the viscosity of the microenvironment.

It is interesting to note that a similar behavior can be highlighted at both the two temperatures investigated, although the effect is higher at 37 °C. As expected, the viscosity of all the systems reduces on increasing the temperature, in agreement with previous measurements [26]. The addition of CDs increases the viscosity of the bilayer, but, on increasing the CD concentration, the viscosity reduces. In particular at 25 °C the viscosity at the highest investigated CD concentrations becomes lower than that of the pure bilayer, whereas at 37 °C even at the highest concentrations of CD investigated, the viscosity reaches a value higher than that of the pure bilayer.

These pieces of evidence suggest interactions of the CDs with the liposomes. In particular, strongly hydrogen bonding CDs, i.e., mainly β-CD and, to a lesser extent, DACD, may favor superficial interactions of the CDs with the phospholipidic head groups that may yield a corresponding reorganization of the phospholipidic hydrocarbon chains that resembles a sort of gel-like phase. These data are in agreement with previously published DSC data evidencing depression of the pretransition peak (L_β_→P_β_) of DPPC vesicles and higher T_m_ values of the main transition peak (P_β_→L_α_) of DPPC vesicles in the presence of different CDs, with respect to that for pure DPPC dispersions. Both these effects were imputed to hydrogen bonding between CDs and phospholipidic bilayer′s head groups [36]. On the other hand the decreased viscosity observed at the highest investigated CDs concentrations, particularly evident in the case of highly OH-rich β-CDs at 25° C, may be imputed to the fact that once the number of hydrogen bond interactions reaches a certain threshold, the structuring of CDs involves an out-and-out stabilizing effect with reduction of the spontaneous pore formation and a promotion of the encounter of pyrene molecules in the bilayer. Indeed, the same effect could be imputed to specific interactions of functionalized CDs with the distal region of the hydrophobic chains of the bilayer; nevertheless, this hypothesis should be discarded as the same effect was observed for nonfunctionalized CDs. 

### 2.3. Liposome Stability

**Figure 2:**
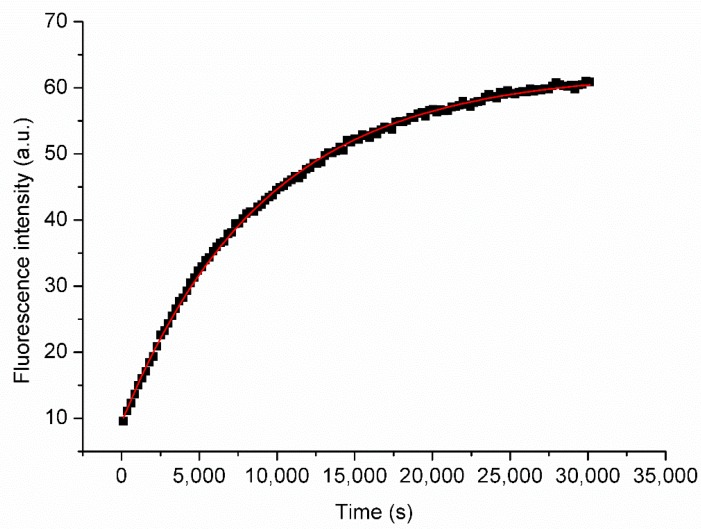
Representative kinetic profile of the release of CF^3−^ from POPC/β-CD liposomes at POPC/β-CD = 5 and 25° C. The red curve is the fitting curve for a first order kinetics.

The kinetic stability of the POPC liposomes was evaluated at two different temperatures (25.0 ± 0.1 °C and 37.0 ± 0.1 °C) and three different POPC/CD molar ratios (12, 5, and 2.5) by investigating the time-dependent leakage of the 5(6)-carboxyfluorescein (CF^3−^) [44] anionic dye from the liposomal aqueous core. The high self-quenching concentration of CF^3−^ used for the hydration of the phospholipidic thin film ensures very low initial fluorescence intensity. When liposomes start to release the dye, the dilution-dependent dequenching of CF^3−^ causes an increase of the fluorescence intensity that is consistent with first-order kinetics (Figure 2, and Supplementary Materials). Therefore, for each breakdown experiment, in the absence or in the presence of CDs, an apparent first-order rate constant *k*_obs_ can be determined according to the following Equation (1).

d[CF^3−^]/dt = *k*_obs_[CF^3−^] (1)

The rate constants, *k*_obs_, for the investigated liposomal systems are reported in Table 3. The most accredited mechanism of leakage of CF^3−^ from POPC liposomes, initially proposed by Kashchiev and Exerova [45], is that the leakage is due to the spontaneous formation in the liposomal bilayer of pores or defects. 

A simple diffusion (dissolution) of the probe into the bilayer is very unlikely because CF^3−^ is nearly insoluble in the hydrophobic bilayer. It has been demonstrated that changing the composition of the bilayer affects its chemicophysical properties and, subsequently, the rate of the release of CF^3−^ [26,38,46,47,48,49,50,51].

The data on CF^3-^ leakage are in perfect agreement with viscosity and dimensional evidence. The addition of functionalized CDs determined mainly a small stabilizing effect for the highest concentrations of CD investigated. This is in agreement with the above mentioned stabilizing effect due to the reduction of spontaneous pore formation associated to the strong interactions between the CDs and the head groups of the phospholipids. The small destabilizing effect observed on addition of β-CD and low concentrations of functionalized CDs may be imputed to the rearrangement consequent to the partial restructuring of CD’s on the liposomal surface. No evidence of penetration of CDs in the liposomal bilayer or extraction of phospholipid to form POPC–CD complexes has been observed. The obtained data are in agreement with a previous study [27] in which POPC SUV liposomes demonstrated to be relatively stable upon the addition of methyl and hydroxypropyl-β-CD (HP-CD) as compared to MLV. Only at very high concentrations of methylated CDs (i.e., 50 times the concentration of phospholipids) SUVs stability was affected, likely due to the extraction of phospholipids (PL) and formation of the relevant complexes. The authors speculated that the curvature of the lipid membrane of substantially smaller SUVs (compared to MLV) did not allow functionalized CD molecules to establish the contact angle needed for lipid extraction from the lipid bilayer. A similar conclusion can be drawn from stability data of MLV in the presence of β-CD [26] and the present data. As a matter of fact, the addition of β-CD to POPC MLVs renders the liposomes ca. 2 times less stable. On the other hand, in the present case the addition of β-CD to POPC SUVs at the highest concentration of β-CD investigated (i.e., the POPC/CD ratio is 0.65 [26]) does not alter the leakage rate of SUVs at 37 °C.

### 2.4. Molecular Dynamics Simulation

**Figure 3:**
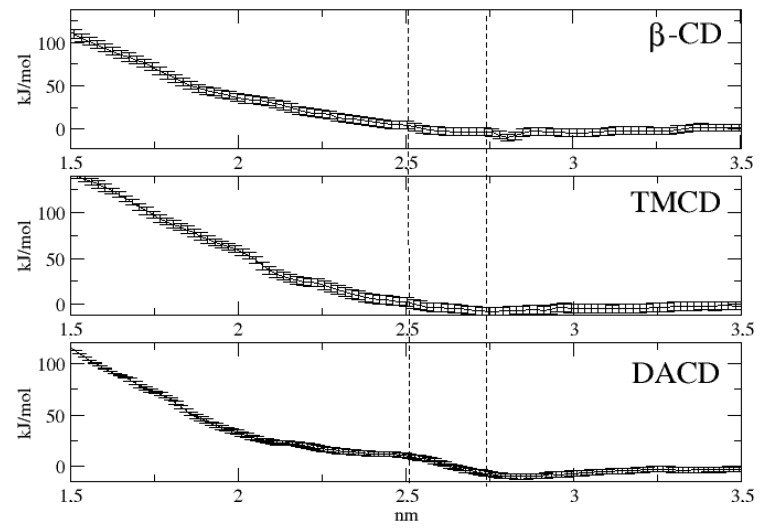
Free energy profile at 298 K as a function of the distance between cyclodextrin and bilayer centers of mass.

**Figure 4:**
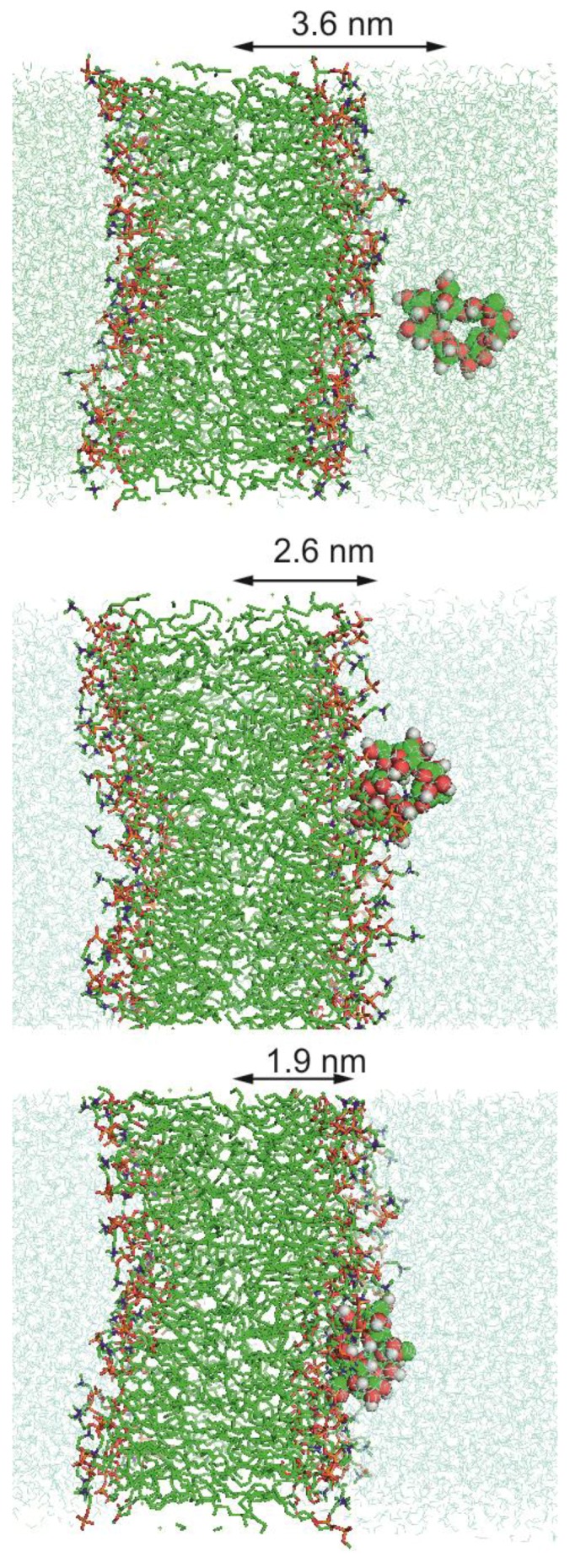
Snapshots taken from US calculations representing three different distances between β-CD and bilayer.

As already reported in the introduction, US calculations were performed with the only aim of estimating the energetics associated to the insertion of each of the three investigated cyclodextrins into the lipid bilayer mimicking the liposome surface. In Figure 3, we report the free energy at 298 K as a function of the direction normal to lipid bilayer whose center of the mass is set to zero. For the sake of clarity we have also reported, in the same figure with vertical dotted lines, the range spanned by the membrane surface along the simulations and, moreover, we have also represented, in Figure 4, three different cyclodextrin–membrane distances taken as snapshot along the simulations.

The obtained free energy profiles turn out to be quite similar in all the cases. In fact, the three cyclodextrins appear to approach the membrane through a diffusion-like motion up to approximately 2.4 nm, i.e., in correspondence of the membrane surface. Subsequently, the free energy profiles appear to undergo a sudden increase, reaching values much larger than 180 kJ/mol at the center of the bilayer. This latter result clearly indicates that, according to our data and in line with previous computational studies [52], penetration into the membrane represents a process practically impossible both by thermodynamic and kinetic point of view. In other words all the three investigated cyclodextrins, because of the favorable interactions with the POPC heads are expected to mainly reside on the liposome surface.

## 3. Conclusions

Functionalized CDs were embedded into POPC liposomes at different concentrations and the obtained colloidal systems were characterized from various points of view. Dimensions slightly increase on addition of CDs and the increase is proportional to the concentration of TMCD and DACD, whereas no size change was recorded in the presence of different concentration of native β-CD. The viscosity and stability data demonstrate that at the investigated concentrations of CDs, independently of the functionalization, a supramolecular structuring of the CDs around the phospholipidic head groups via hydrogen bonding and van der Waals interactions ensures a stabilization of the liposomes, with reduction of the spontaneous pore formation and a consequent promotion of excimer formation. Molecular dynamic simulations confirm that the CDs have no tendency to enter the lipid bilayer.

## 4. Experimental Section

### 4.1. Materials

1-Palmitoyl-2-oleoyl-*sn*-glycero-3-phosphocholine (POPC) was purchased from Avanti Polar Lipids (Alabaster, AL, USA). CF^3−^ (95% purity) and Sephadex G-25 were purchased from Merck KGaA (Darmstadt, Germany). Native β-cyclodextrin (β-CD) was purchased as CAVAMAX^®^ W7 PHARMA BETADEX from Wacker Chimie S.A.S. (Lyon, France). Heptakis(2,3,6-tri-*O*-methyl)-β-cyclodextrin (TMCD) was synthesized according to a previously described method [32]. Heptakis(2,3-di-*O*-acetyl)-β-cyclodextrin (DACD) was prepared following previously described methods [34,35]. β-CD, TMCD, and DACD were dried under vacuum at 80 °C for 48 h prior to use.

### 4.2. Instruments

The extrusion was performed on a nitrogen-driven Lipex Biomembranes (Vancouver, BC, Canada) apparatus operating at room temperature. UV/Vis absorption measurements were performed on Jasco V-550UV/Vis (Cremella, Italy). Luminescence intensity measurements were performed with Jasco FP-6200 and Jasco FP-6500 spectrofluorimeters. Measurements of vesicle size were performed by using a 90Plus/BI-MAS ZetaPlus multiangle particle size analyzer (Brookhaven Instruments Corp., Holtsville, NY, USA) on dilute samples. The osmolarity of the solutions was checked using a micro-osmometer (Advanced Instruments Model 3300, Norwood, MA, USA), while the pH of the solutions was checked on a pHM93 pH meter from Radiometer (Brønshøj, Denmark). 

### 4.3. POPC Liposome Preparation

An appropriate amount of a stock solution of POPC in chloroform (25 mg mL^−1^) was evaporated at 40 °C under reduced pressure by rotary evaporation to form a thin phospholipid film on the inside wall of a flask. The thin lipid film formed was vacuum dried and kept at 4 °C overnight before rehydration with an aqueous buffer solution containing cyclodextrins in order to obtain phospholipid/cyclodextrin molar ratios of 12:1; 5:1, and 2.5:1. 

The resulting multilamellar vesicle dispersions were extruded five times through polycarbonate filters with 100-nm pores [26,38,48,49,50,51].

For quenching/dequenching measurements, the rehydration of the lipidic film was performed by using 50 mM CF^3−^ buffered aqueous solution. To get rid of unentrapped dye the liposomes were passed through a Sephadex G-25 column. Extrusion and gel permeation column chromatography were performed at room temperature. 

Prior to use, the liposomal solutions were diluted with the appropriate buffer (isosmotic to the one used during the rehydration step) to give a final concentration of POPC equal to 1.32 × 10^−5^ M for stability measurements and 2.64 × 10^−4^ M for viscosity measurements, assuming 100% elution of the lipid during the gel filtration process.

The buffer (pH 7.4) used for the stability measurements was made of 121.5 mM NaCl, 25.2 mM Na_2_HPO_4_, 4.8 mM KH_2_PO_4_, and 50 mM CF^3−^. The buffer (pH 7.4) used for viscosity measurements was made of 121.5 mM NaCl, 25.2 mM Na_2_HPO_4_, and 4.8 mM KH_2_PO_4_.

### 4.4. Fluorimetric Measurements

The viscosity and the polarity of the liposomal membrane have been fluorimetrically determined at two different temperatures (25.0 ± 0.1 °C and 37.0 ± 0.1 °C) using pyrene as the fluorescent probe. The fluorescence intensity ratio, I_E_/I_M_, was used [41] for membrane microviscosity estimation (were I_E_ stays for fluorescence intensity of the excimer at λ = 480 nm and I_M_ for fluorescence intensity of the pyrene monomer at λ = 395 nm). Indeed, the relative intensities of excimer and monomer fluorescence of pyrene have been shown to be a simple linear function of the viscosity of a homologous series of solvents [53,54]. This relationship was demonstrated to be valid as well for micellar [53] and liposomal systems [54] and may be represented by Equation (2).

C′_h_ = (I_M_/I_E_) × C = 1/K × η (2)

where C is the concentration of pyrene, K is a constant that depends on the temperature, and η is the viscosity. C′_h_ can therefore be obtained as the reciprocal of the slope of the plot of I_E_/I_M_ versus the concentration of pyrene, C. Fluorescence emission spectra were taken at the excitation wavelength λ_ex_ = 335 nm. The final concentrations of POPC, pyrene, and CDs in the final liposomal suspension were 2.64 × 10^−4^ M, 1 × 10^−5^ M, and 2.2 × 10^−5^ M, 5.28 × 10^−5^ M and 1.06 × 10^−4^ M for POPC/CD ratio 12, 5, and 2.5, respectively. 

Stability measurements were made monitoring CF^3−^ leakage from liposome at 25.0 ± 0.1 and 37.0 ± 0.1 °C. The fluorescence intensity was measured at an emission wavelength of 516 nm using λ_ex_ = 490 nm as the excitation wavelength. 

### 4.5. Molecular Dynamics Simulations

Cyclodextrins have been extensively studied through MD simulations. For this reason we have based our computational setup on the available literature [55,56,57,58,59]. All simulations were carried out using the Gromacs package [60,61] version 5.0.2 (Science for Life Laboratory, Stockholm, Sweden). The three investigated cyclodextrins, i.e., β-CD, TMCD, and DACD, whose structure was constructed with the program Molden [62] and minimized with the same level of calculations utilized for calculating the atomic charges, were modeled by adapting the Gromos force field (gromos53a6) [63] with the atomic charges calculated ex novo using standard fitting procedures with the program Gaussian 09 [64] in the framework of Density Functional Theory with the CAM-B3LYP functional [65] in conjunction with the 6-31G* basis set. Details of the cyclodextrins topologies are reported in the Supplementary Materials.

For the lipid bilayer (POPC) and water representation we adopted the force field taken from the literature [66] and the Simple Point Charge (spc) model [67], respectively. The simulations were then performed in the NVT ensemble with a time step of 2.0 fs adopting a rather standard protocol: after an initial energy minimization, the system was gradually heated from 50 to 250 K using short (20 ps) MD simulations. Finally a further preequilibration of the system, arrived at 298K, was carried out by running 1.0 ns of MD simulation in all the systems. The temperature was kept constant using the velocity rescaling procedure [68]. The LINCS algorithm was employed to constraint all bond lengths [69]. Long-range electrostatic interactions were computed by the Particle Mesh Ewald method with 34 wave vectors in each dimension and a fourth-order cubic interpolation, and a cut-off of 1.1 nm was used [70]. 

Determination of the free energy of insertion of the cyclodextrins into the lipid bilayer was carried out using the following procedure.

(i) Generation of initial configurations: at this purpose, we utilized constant velocity-steered MD simulations. The center of mass of the substrate was pulled in the z-dimension (normal to the bilayer) forcing the substrate to move from the solvent (water) toward the middle of the membrane. A harmonic potential with a force constant of 1000 kJ mol^−1^ nm^−2^ was adopted in conjunction with a center of mass constant velocity of 0.001 nm ps^−1^. 

(ii) Umbrella sampling: The umbrella sampling (US) procedure [71] with the weighted histogram analysis method (WHAM) [72] was performed with a harmonic potential of 3000 kJ mol^−1^ nm^−2^ in the z direction between the center of mass of the substrate and the center of mass of the POPC bilayer. Twenty windows separated by 0.15 nm were produced and independently simulated for 10 ns. The error was evaluated through standard bootstrap analysis [73].

## Figures and Tables

**Table 1:** Size of pure and CD-doped 1-palmitoyl-2-oleoyl-*sn*-glycero-3-phosphocholine (POPC) liposomes measured at 25.0 ± 0.1 °C.

Entry	Liposome Composition	POPC/CD ^[a]^	Diameter (nm)	Polydispersity
1	POPC		118.0 ± 0.5	0.02 ± 0.01
2	POPC/β-CD	12	131.6 ± 1.5	0.05 ± 0.01
3	POPC/β-CD	5	130.5 ± 2.3	0.05 ± 0.02
4	POPC/β-CD	2.5	132.2 ± 0.7	0.08 ± 0.01
5	POPC/TMCD	12	128.6 ± 4.7	0.18 ± 0.01
6	POPC/TMCD	5	136.6 ± 1.0	0.13 ± 0.01
7	POPC/TMCD	2.5	144.1 ± 3.0	0.11 ± 0.01
8	POPC/DACD	12	144.6 ± 2.3	0.11 ± 0.02
9	POPC/DACD	5	160.7 ± 4.2	0.19 ± 0.03
10	POPC/DACD	2.5	185.8 ± 0.6	0.29 ± 0.01

[a] Molar ratio.

**Table 2:** Bilayer viscosity (C′_h_) ^[a]^ in pure and CD-doped POPC liposomes.

Entry	Liposome Composition	POPC/CD ^[b]^	C′_h_ × 10^5^ (M) at 25.0 ± 0.1 °C	C′_h_ × 10^5^ (M) at 37.0 ± 0.1 °C
1	POPC		13.80 ± 0.10	4.52 ± 0.01
2	POPC/β-CD	12	11.70 ± 0.40	8.31 ± 0.58
3	POPC/β-CD	5	7.44 ± 0.11	5.37 ± 0.15
4	POPC/β-CD	2.5	7.16 ± 0.08	6.14 ± 0.15
5	POPC/TMCD	12	14.40 ± 0.40	11.70 ± 0.10
6	POPC/TMCD	5	13.90 ± 0.05	9.37 ± 0.20
7	POPC/TMCD	2.5	6.75 ± 0.54	5.20 ± 0.18
8	POPC/DACD	12	15.10 ± 0.60	11.00 ± 0.90
9	POPC/DACD	5	15.80 ± 1.50	11.00 ± 0.09
10	POPC/DACD	2.5	8.07 ± 0.20	5.76 ± 0.11

[a] C′_h_ is the reciprocal of the slope of the plot of I_E_/I_M_ vs. pyrene concentration; [b] Molar ratio.

**Table 3:** First-order rate constants for the release of carboxyfluorescein (CF^3−^) from pure and CD-doped POPC liposomes.

Entry	Liposome Composition	POPC/CD ^[a]^	10^−4^ *k_obs_* (s^−1^) at 25.0 ± 0.1 °C	10^−4^ *k_obs_* (s^−1^) at 37.0 ± 0.1 °C
1	POPC		1.31 ± 0.05	0.90 ± 0.04
2	POPC/β-CD	12	1.18 ± 0.05	1.15 ± 0.05
3	POPC/β-CD	5	1.18 ± 0.13	1.40 ± 0.19
4	POPC/β-CD	2.5	0.28 ± 0.03	0.93 ± 0.02
5	POPC/TMCD	12	1.48 ± 0.05	1.24 ± 0.06
6	POPC/TMCD	5	0.89 ± 0.03	0.60 ± 0.03
7	POPC/TMCD	2.5	0.32 ± 0.07	0.33 ± 0.08
8	POPC/DACD	12	1.40 ± 0.15	1.54 ± 0.23
9	POPC/DACD	5	1.62 ± 0.15	1.74 ± 0.16
10	POPC/DACD	2.5	0.30 ± 0.05	0.58 ± 0.08

[a] Molar ratio.

## Supplementary Materials

The following are available online, Figures S1–S20: Representative plots of I_E_/I_M_ vs. pyrene concentration for pure POPC, POPC/β-CD, POPC/TMCD and POPC/DACD liposomes at 25 °C and 37 °C, Figures S21–S40: Representative kinetic profiles of the release of CF^3−^ from pure POPC, POPC/β-CD, POPC/TMCD and POPC/DACD liposomes at 25 °C and 37 °C, Cartesian coordinates of β-CD, TMCD and DACD, atom-types and charges for topology of β-CD, TMCD and DACD.
