# Supplementary material for: Effect of the Incorporation of Functionalized Cyclodextrins in the Liposomal Bilayer
Source: Molecules. 2019 Apr 9;24(7):1387. doi: 10.3390/molecules24071387 (PMC6479378; doi:10.3390/molecules24071387)
Supplement: Supplementary file 1 [file molecules-24-01387-s001.pdf]

Article

Effect of the Incorporation of Functionalized Cyclodextrins in the Liposomal Bilayer

Romina Zappacosta [1], Benedetta Cornelio [2], Serena Pilato [1], Gabriella Siani [1], François Estour [2,*], Massimiliano Aschi [3] and Antonella Fontana [1,*]

[1] Dipartimento di Farmacia, Università "G. d'Annunzio", Via dei Vestini snc, I-66100 Chieti, Italy; r.zappacosta@unich.it (R.Z.); serena.pilato@unich.it (S.P.); gabriella.siani@unich.it (G.S.)
[2] Normandie Univ, INSA Rouen, UNIROUEN, CNRS, COBRA (UMR 6014 & FR3038), 76000 Rouen, France; benedettacornelio@hotmail.com
[3] Dipartimento di Scienze Fisiche e Chimiche, Università di L'Aquila, Via Vetoio snc, I-67100 L'Aquila, Italy, massimiliano.aschi@univaq.it
* Correspondence: francois.estour@univ-rouen.fr (F.E.); antonella.fontana@unich.it (A.F.); Tel.: +33-(0)2-3552-2921 (F.E.); +39-0871-3554790 (A.F.)

Supplementary Materials

S1. Viscosity measurements

S2. Stability measurements

S3. Details of Molecular Dynamics Simulations

S1. Viscosity measurements

S1.1 Viscosity measurements for pure POPC liposomes

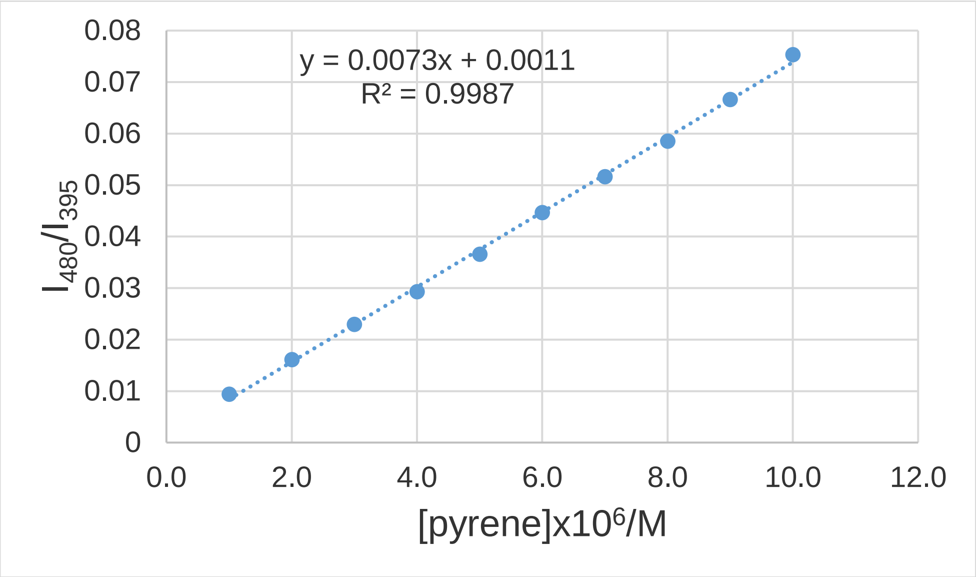

Figure S1. Representative plot of I_E/I_M *vs.* pyrene concentration for pure POPC liposomes at 25° C.

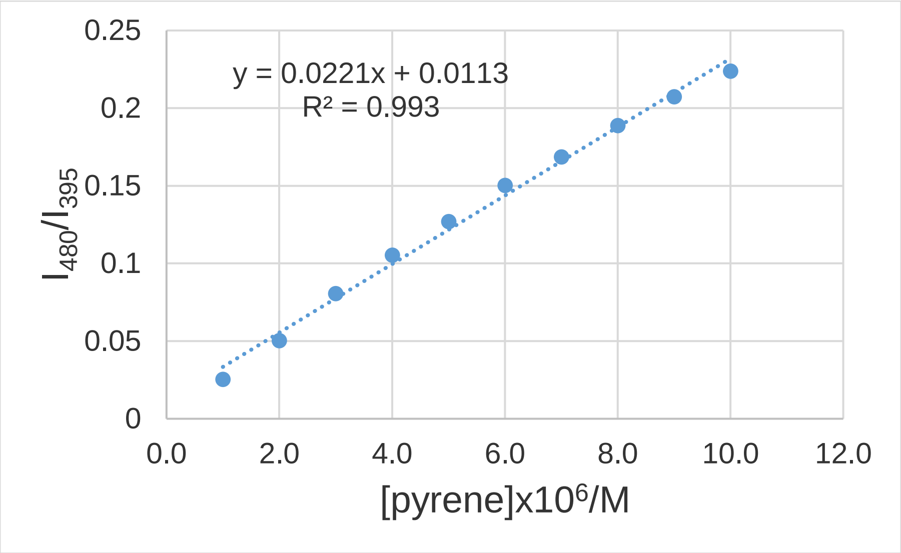

Figure S2. Representative plot of I_E/I_M *vs.* pyrene concentration for pure POPC liposomes at 37° C.

S1.2 Viscosity measurements for POPC/β-CD liposomes

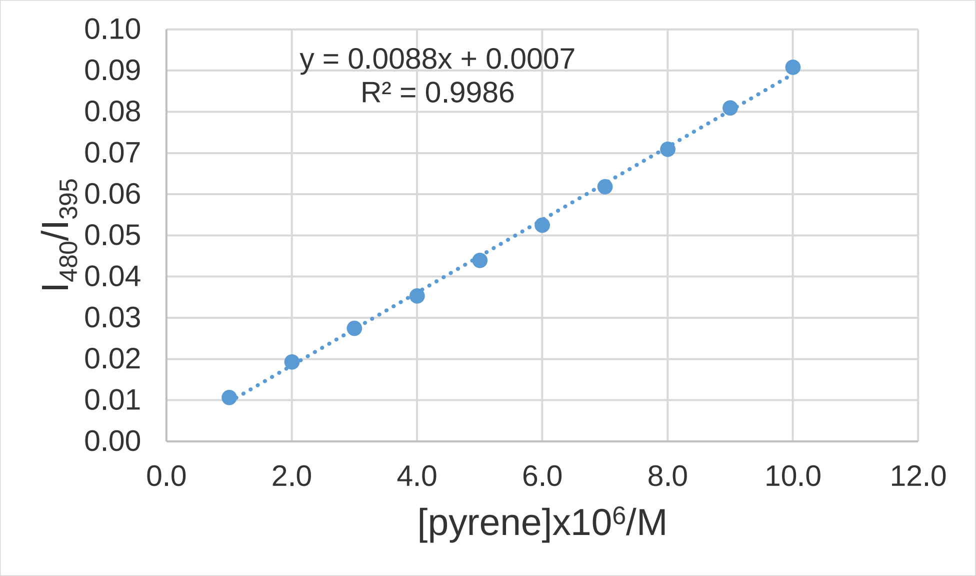

Figure S3. Representative plot of I_E/I_M *vs.* pyrene concentration for POPC/β-CD 12 liposomes at 25° C.

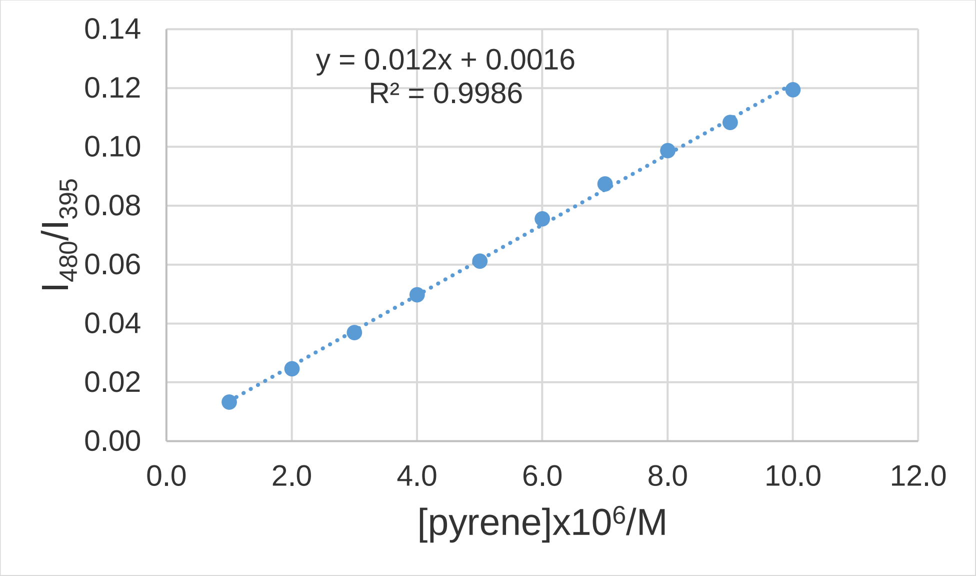

Figure S4. Representative plot of I_E/I_M *vs.* pyrene concentration for POPC/β-CD 12 liposomes at 37° C.

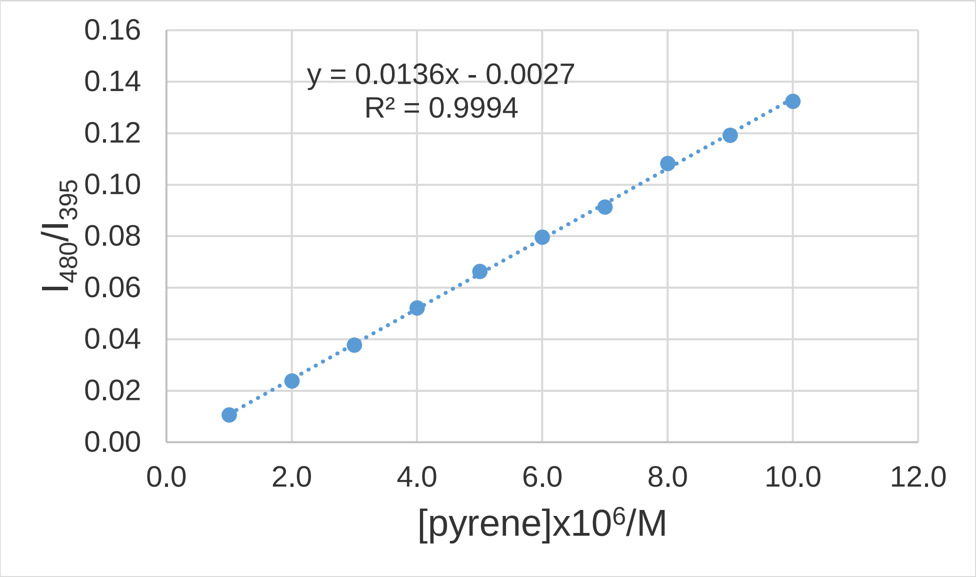

Figure S5. Representative plot of I_E/I_M *vs.* pyrene concentration for POPC/β-CD 5 liposomes at 25° C.

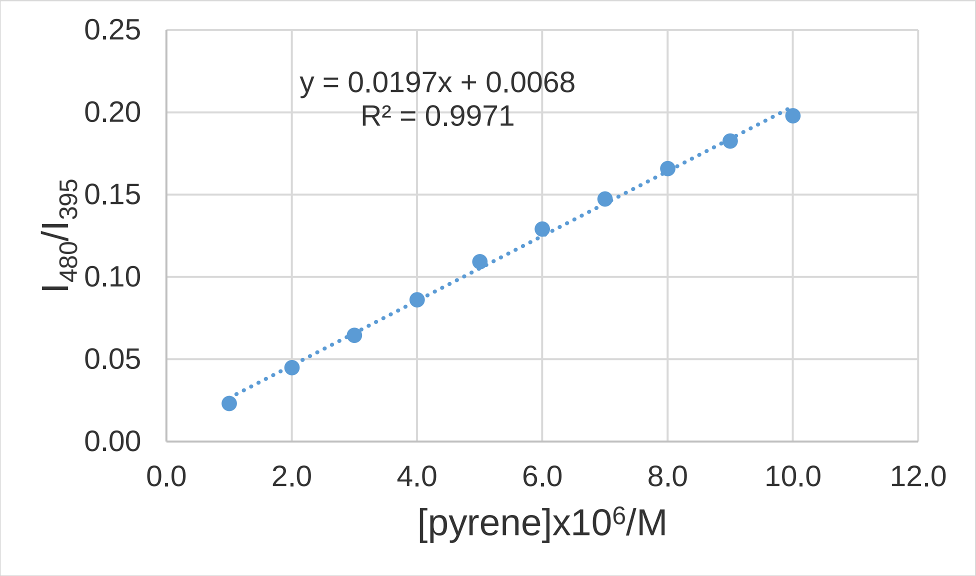

Figure S6. Representative plot of I_E/I_M *vs.* pyrene concentration for POPC/β-CD 5 liposomes at 37° C.

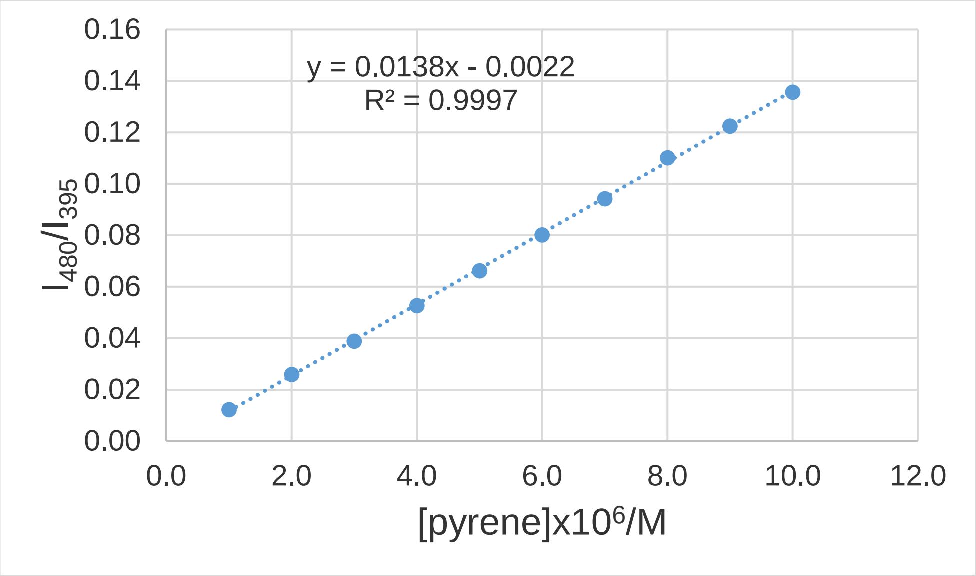

Figure S7. Representative plot of I_E/I_M *vs.* pyrene concentration for POPC/β-CD 2.5 liposomes at 25° C.

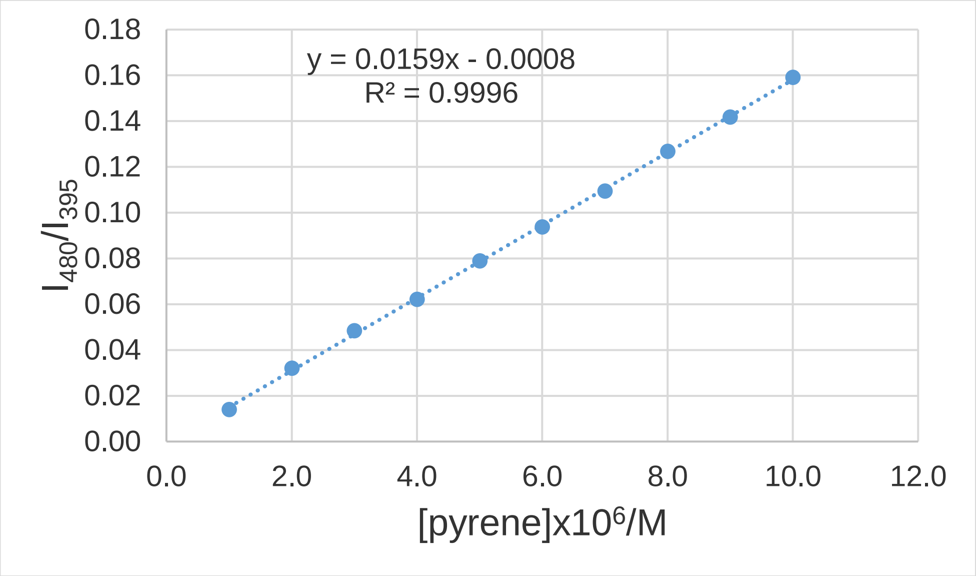

Figure S8. Representative plot of I_E/I_M *vs.* pyrene concentration for POPC/β-CD 2.5 liposomes at 37° C.

S1.3 Viscosity measurements for POPC/TMCD liposomes

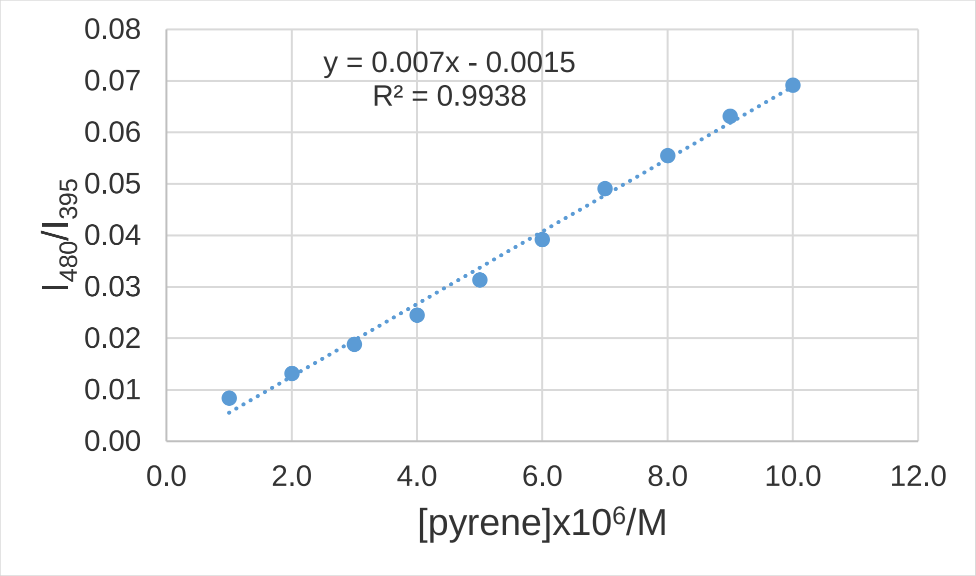

Figure S9. Representative plot of I_E/I_M *vs.* pyrene concentration for POPC/TMCD 12 liposomes at 25° C.

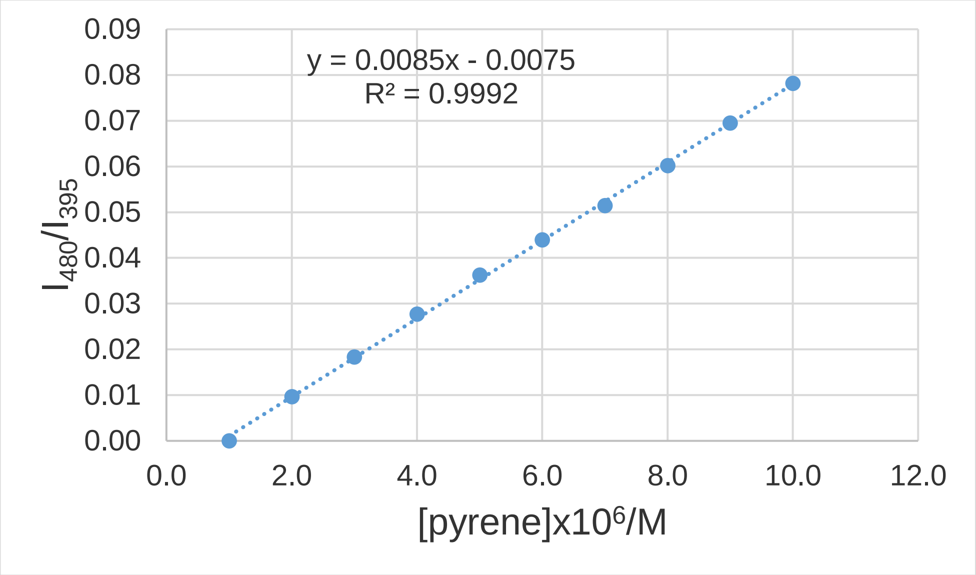

Figure S10. Representative plot of I_E/I_M *vs.* pyrene concentration for POPC/TMCD 12 liposomes at 37° C.

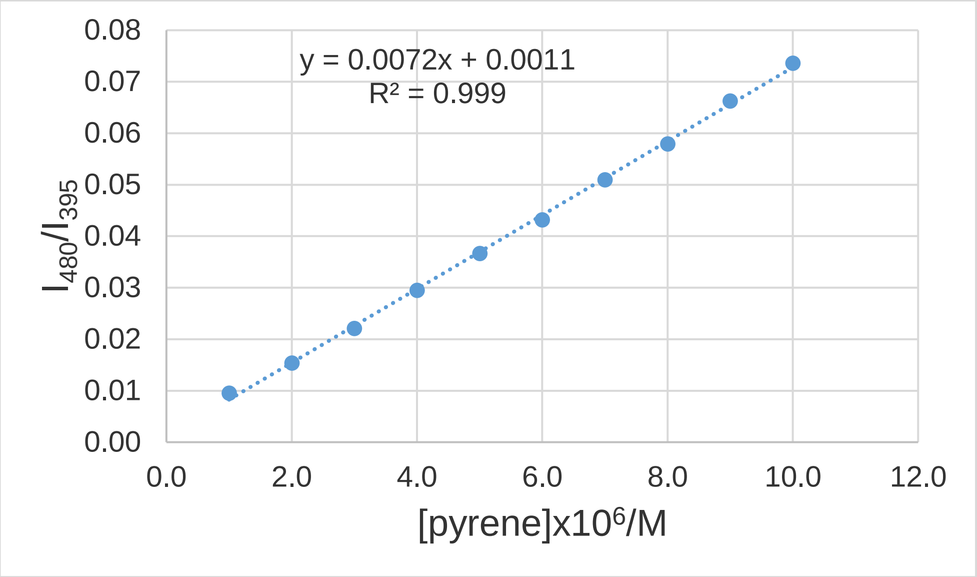

Figure S11. Representative plot of I_E/I_M *vs.* pyrene concentration for POPC/TMCD 5 liposomes at 25° C.

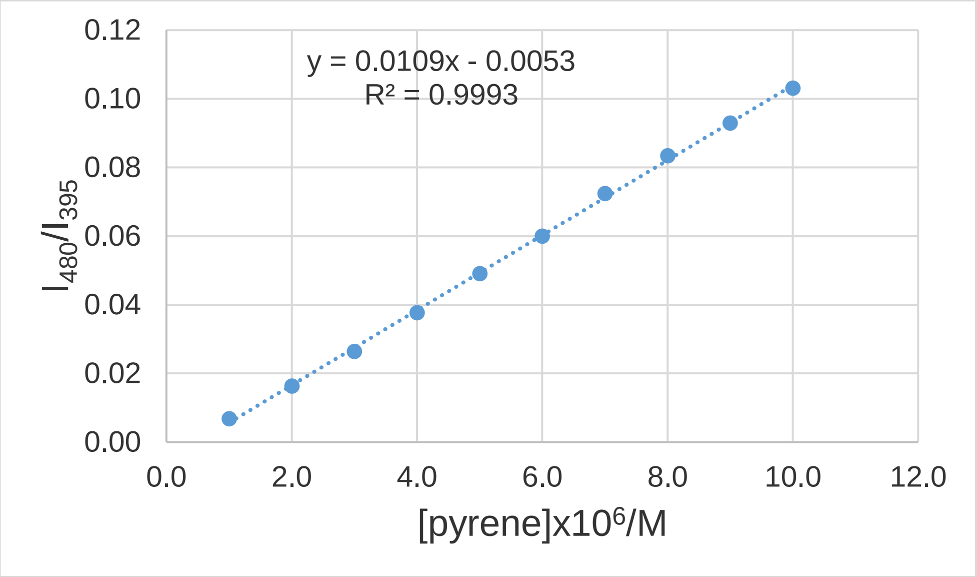

Figure S12. Representative plot of I_E/I_M *vs.* pyrene concentration for POPC/TMCD 5 liposomes at 37° C.

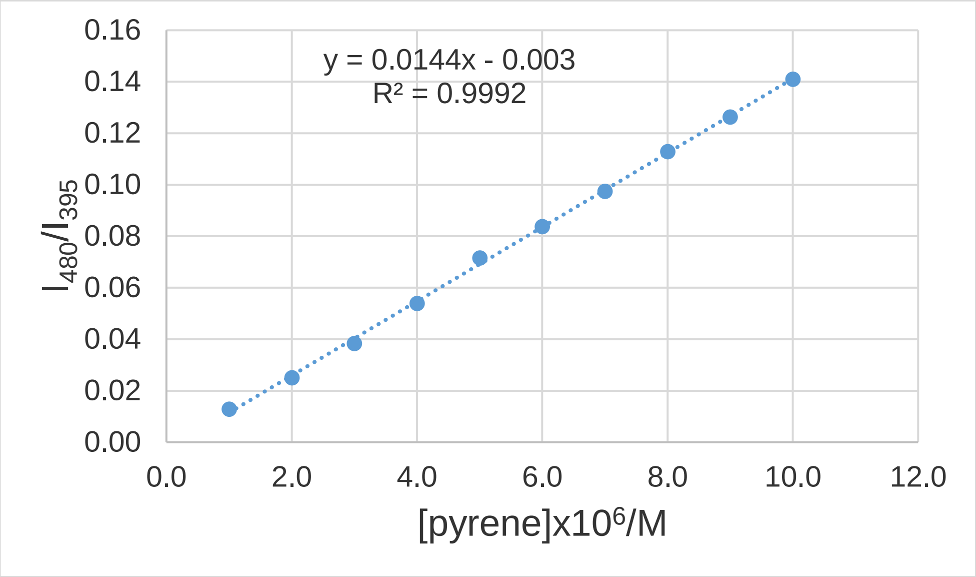

Figure S13. Representative plot of I_E/I_M *vs.* pyrene concentration for POPC/TMCD 2.5 liposomes at 25° C.

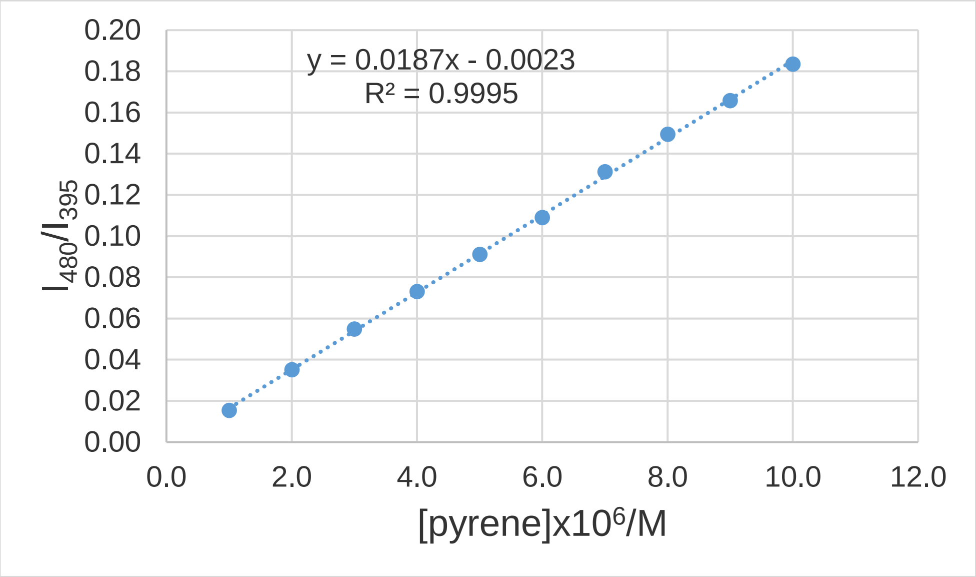

Figure S14. Representative plot of I_E/I_M *vs.* pyrene concentration for POPC/TMCD 2.5 liposomes at 37° C.

S1.4 Viscosity measurements for POPC/DACD liposomes

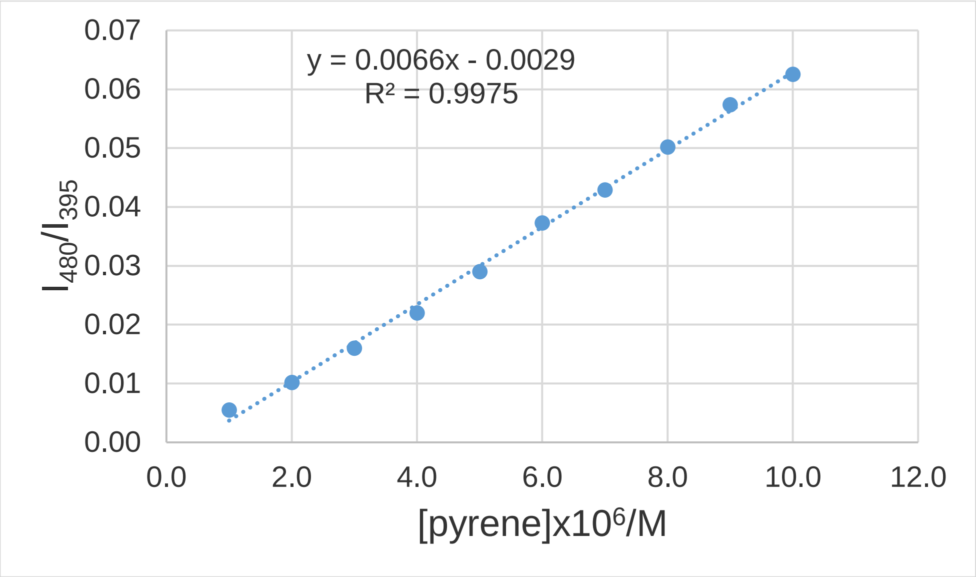

Figure S15. Representative plot of I_E/I_M *vs.* pyrene concentration for POPC/DACD 12 liposomes at 25° C.

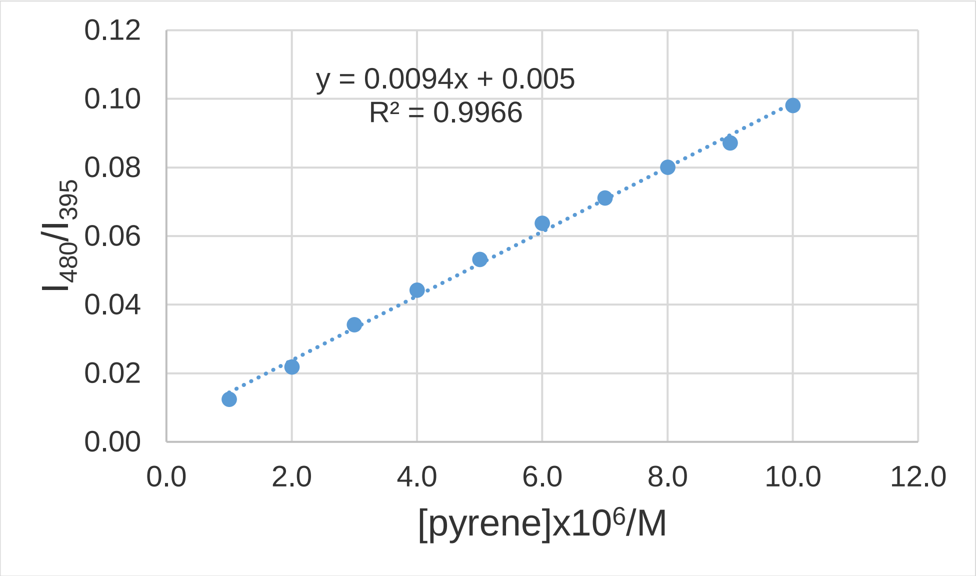

Figure S16. Representative plot of I_E/I_M *vs.* pyrene concentration for POPC/DACD 12 liposomes at 37° C.

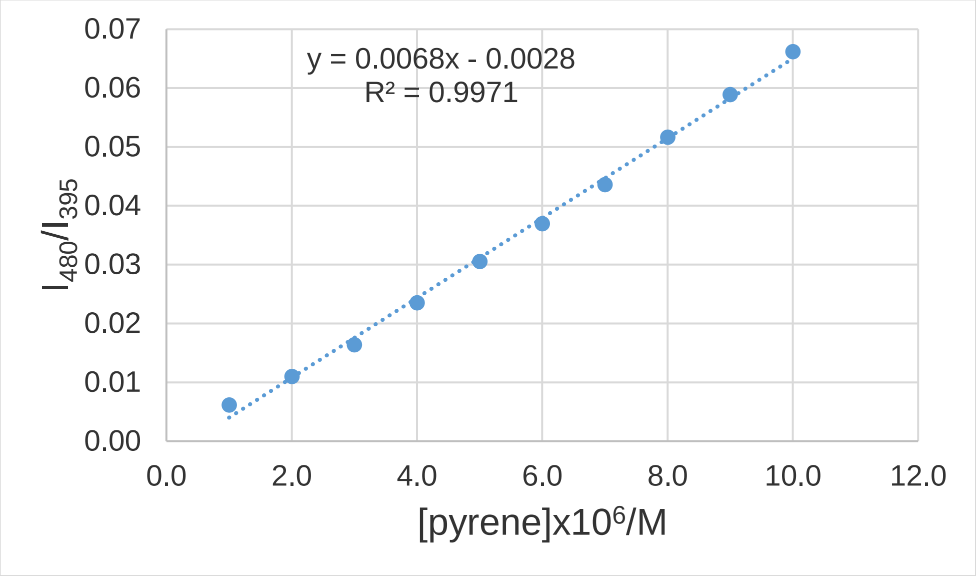

Figure S17. Representative plot of I_E/I_M *vs.* pyrene concentration for POPC/DACD 5 liposomes at 25° C.

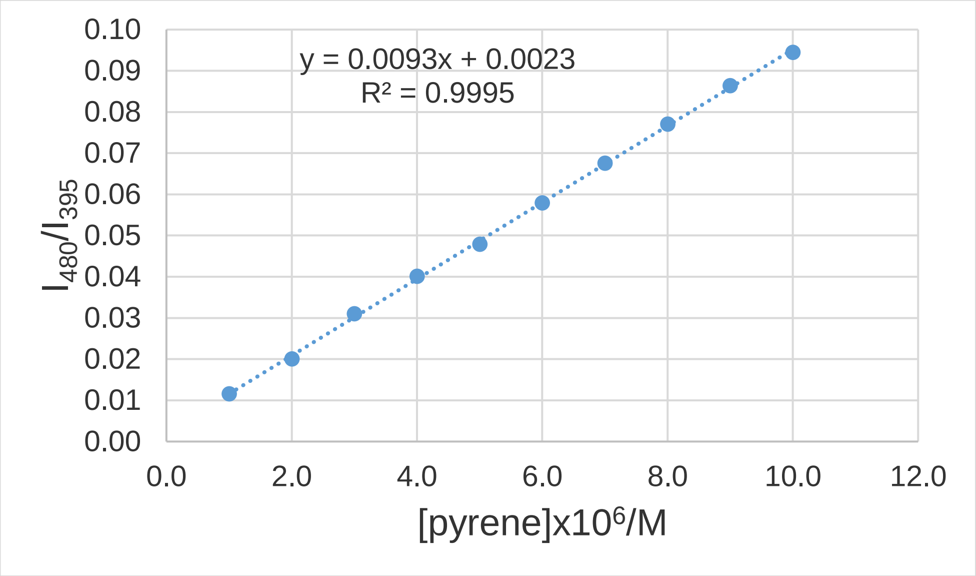

Figure S18. Representative plot of I_E/I_M *vs.* pyrene concentration for POPC/DACD 5 liposomes at 37° C.

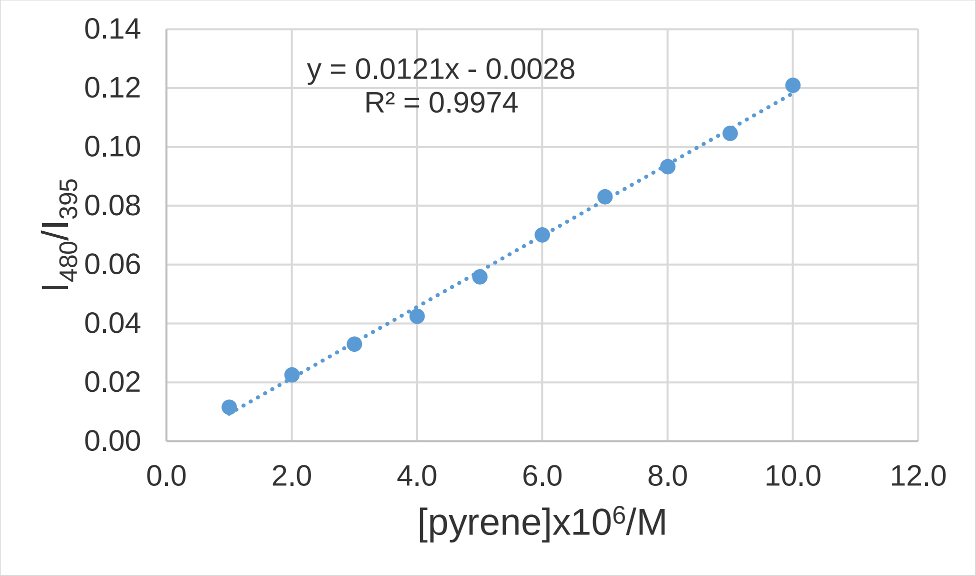

Figure S19. Representative plot of I_E/I_M *vs.* pyrene concentration for POPC/DACD 2.5 liposomes at 25° C.

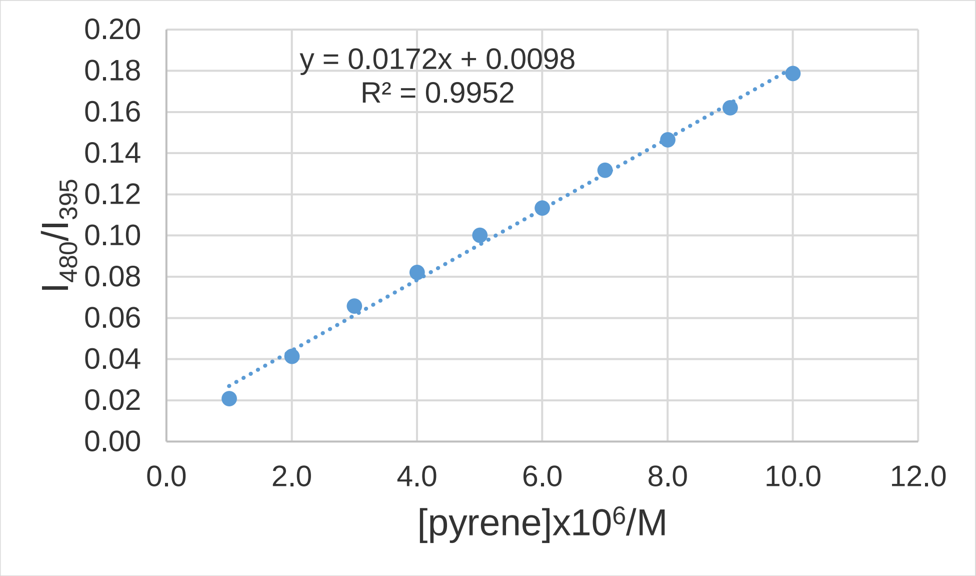

Figure S20. Representative plot of I_E/I_M *vs.* pyrene concentration for POPC/DACD 2.5 liposomes at 37° C.

S2. Stability measurements

S2.1 Stability measurements for pure POPC liposomes

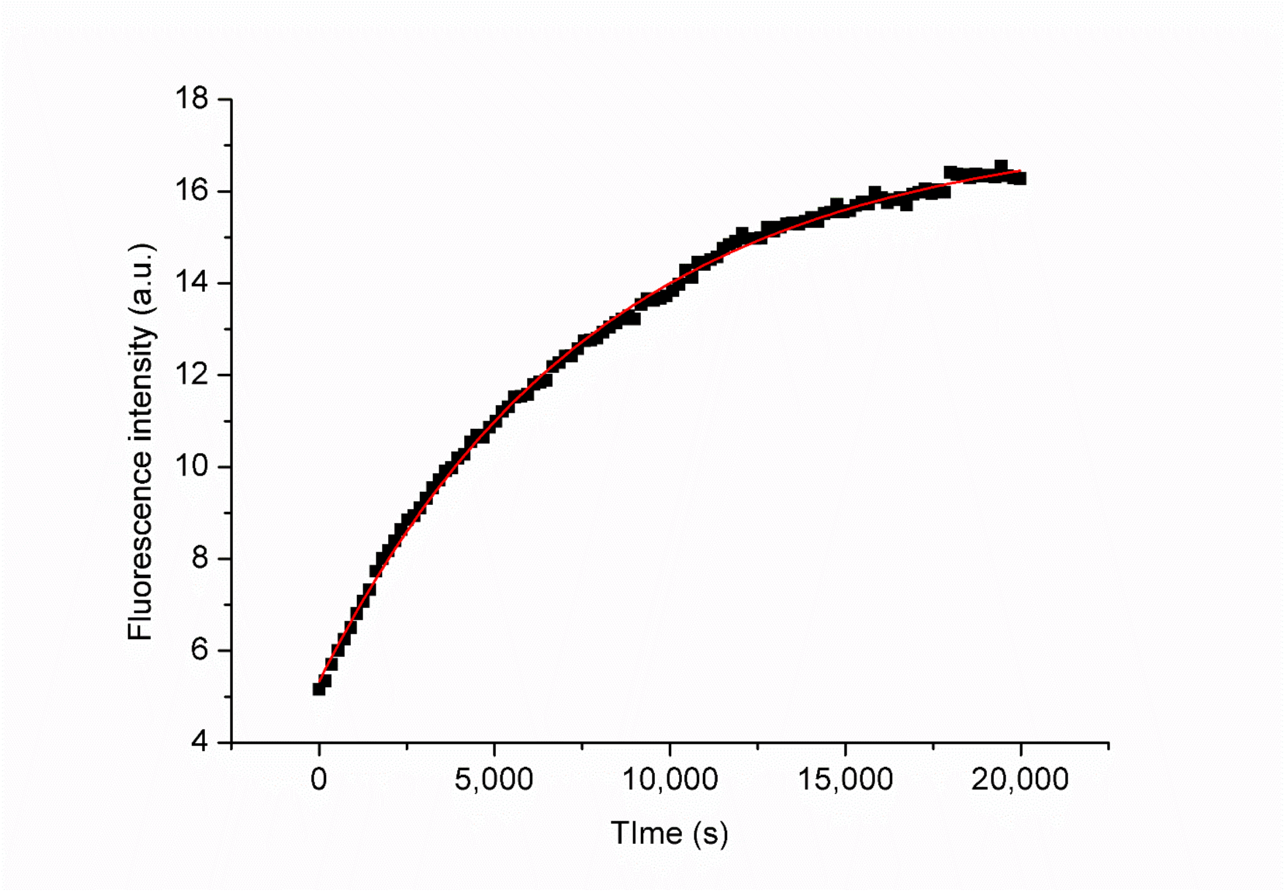

Figure S21. Representative kinetic profile of the release of CF^{3-} from pure POPC liposomes at 25° C.

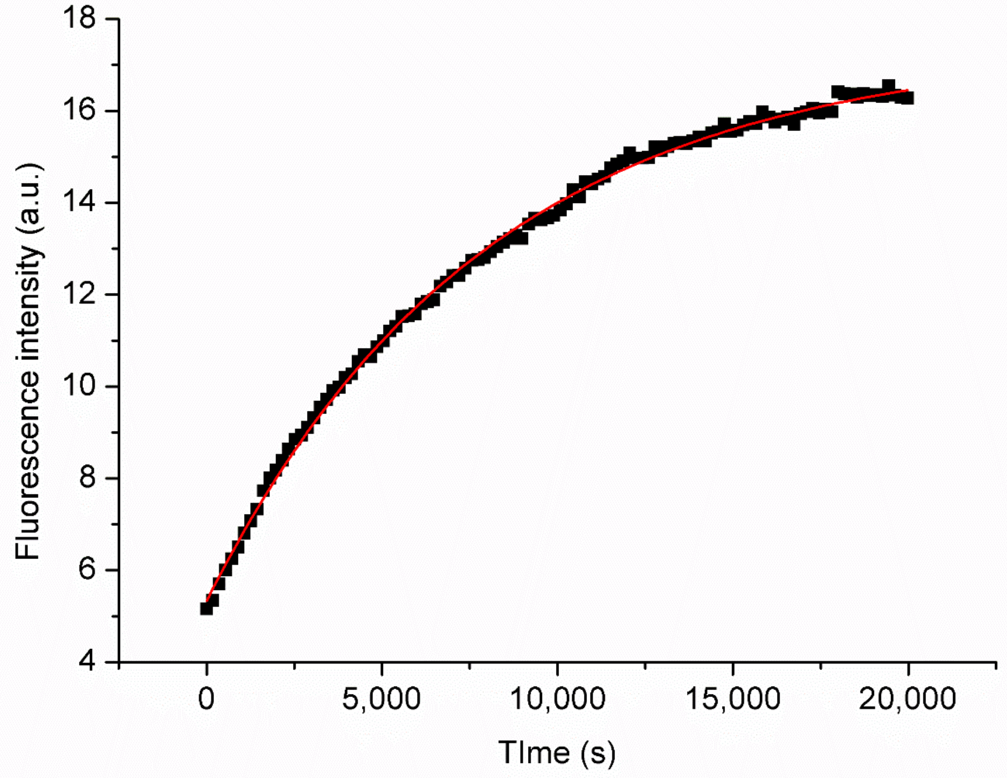

Figure S22. Representative kinetic profile of the release of CF^{3-} from pure POPC liposomes at 37° C.

S2.2 Stability measurements for POPC/β-CD liposomes

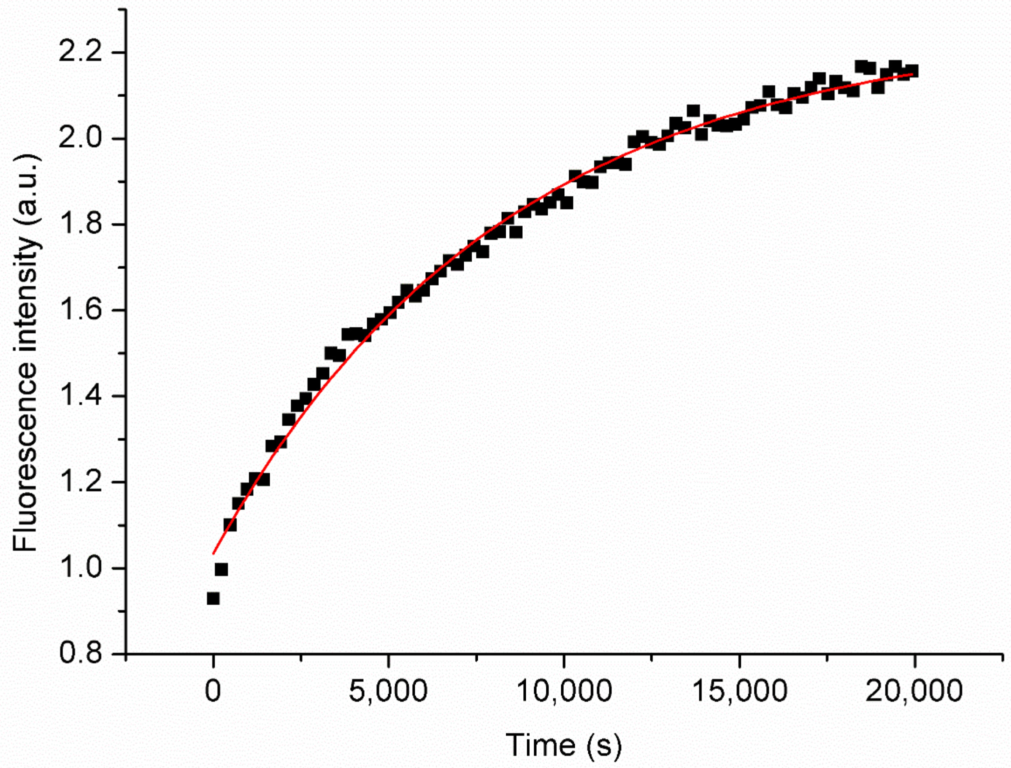

Figure S23. Representative kinetic profile of the release of CF^{3-} from POPC/β-CD 12 liposomes at 25° C.

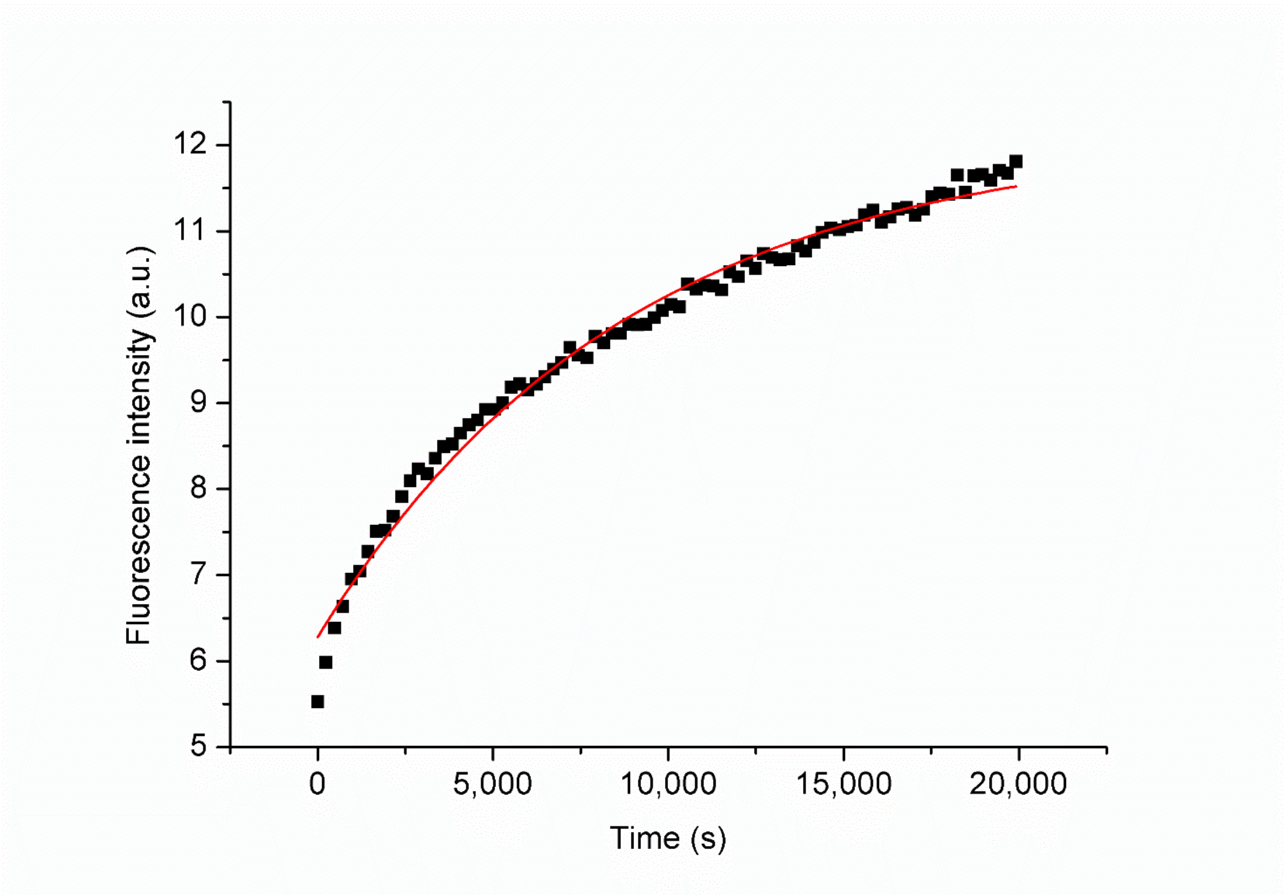

Figure S24. Representative kinetic profile of the release of CF^{3-} from POPC/β-CD 12 liposomes at 37° C.

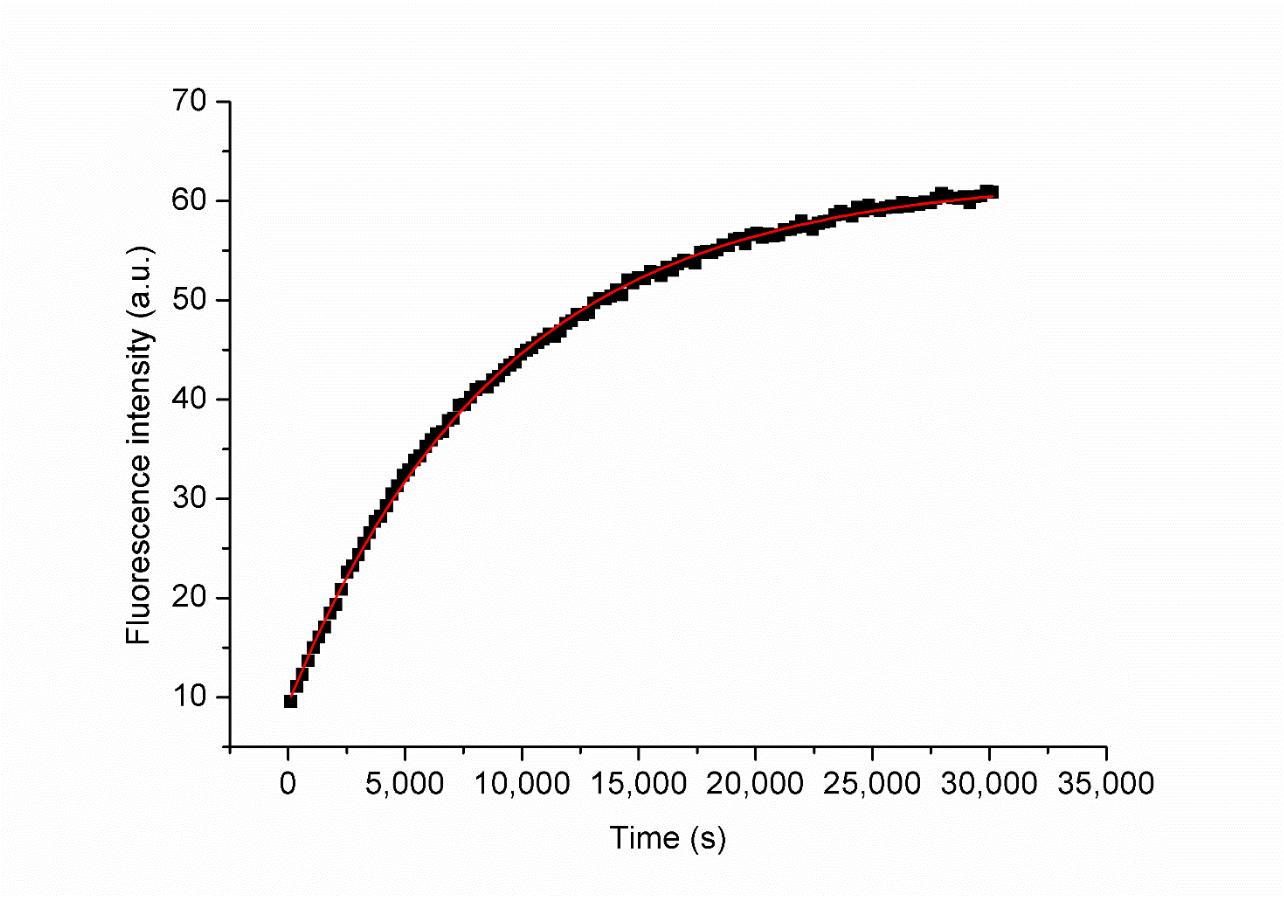

Figure S25. Representative kinetic profile of the release of CF^{3-} from POPC/β-CD 5 liposomes at 25° C.

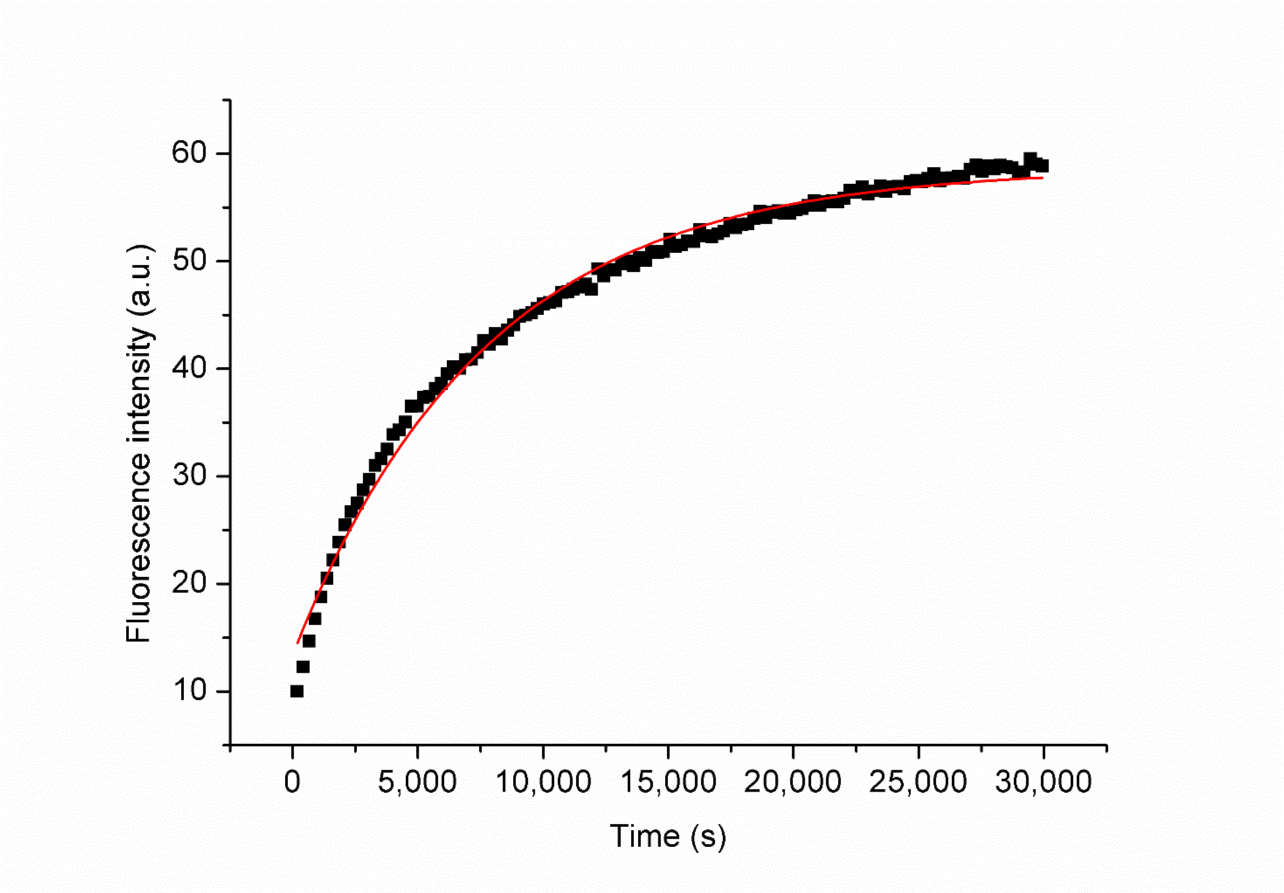

Figure S26. Representative kinetic profile of the release of CF^{3-} from POPC/β-CD 5 liposomes at 37° C.

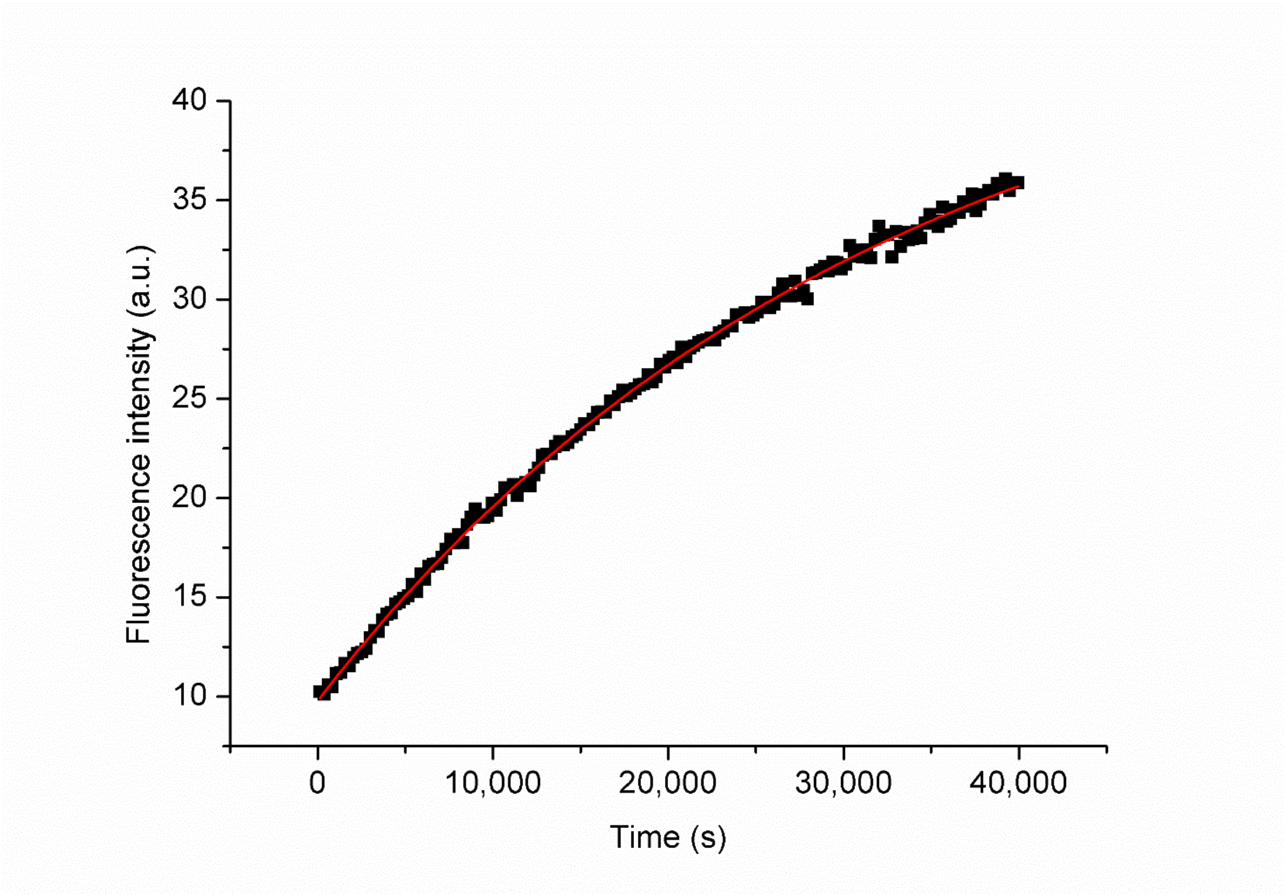

Figure S27. Representative kinetic profile of the release of CF^{3-} from POPC/β-CD 2.5 liposomes at 25° C.

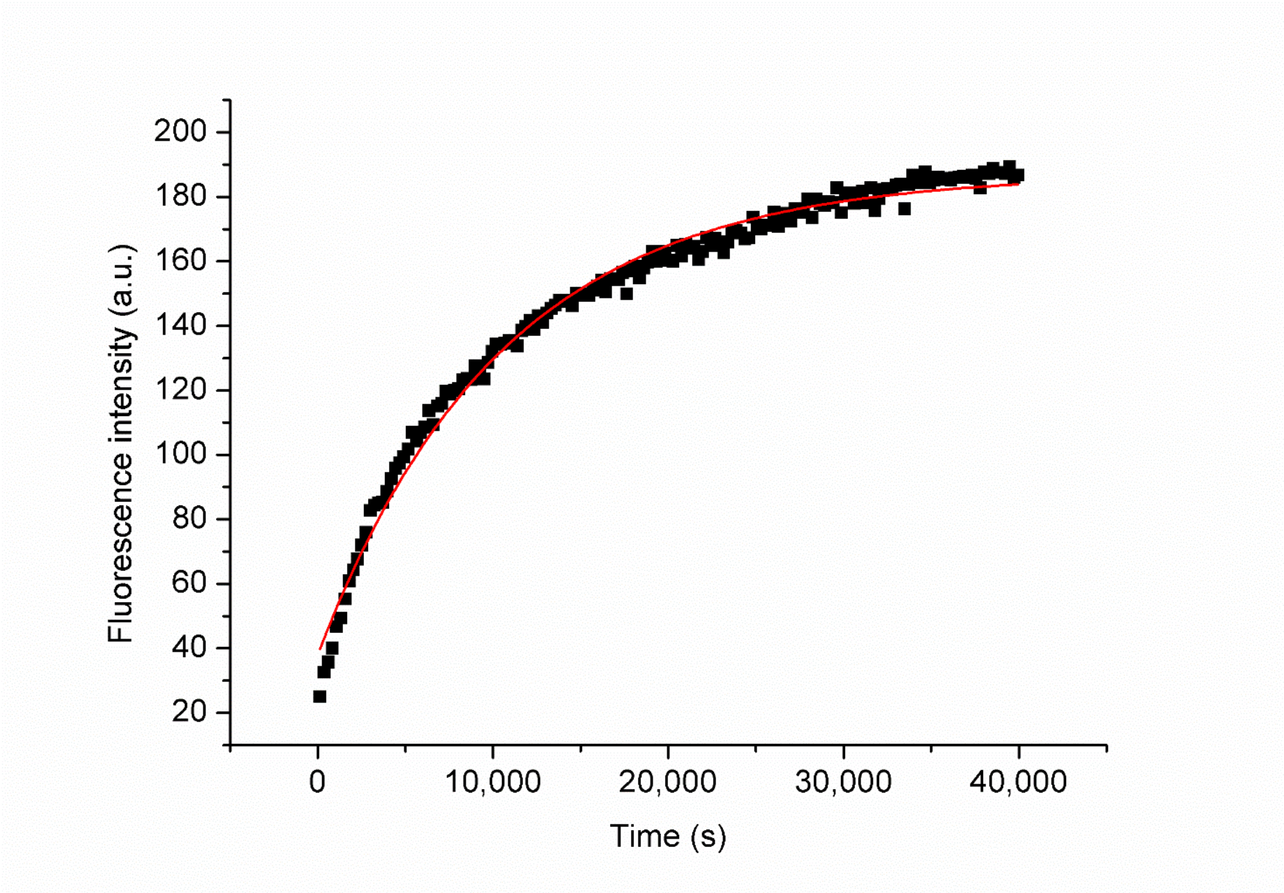

Figure S28. Representative kinetic profile of the release of CF^{3-} from POPC/β-CD 2.5 liposomes at 37° C.

S2.3 Stability measurements for POPC/TMCD liposomes

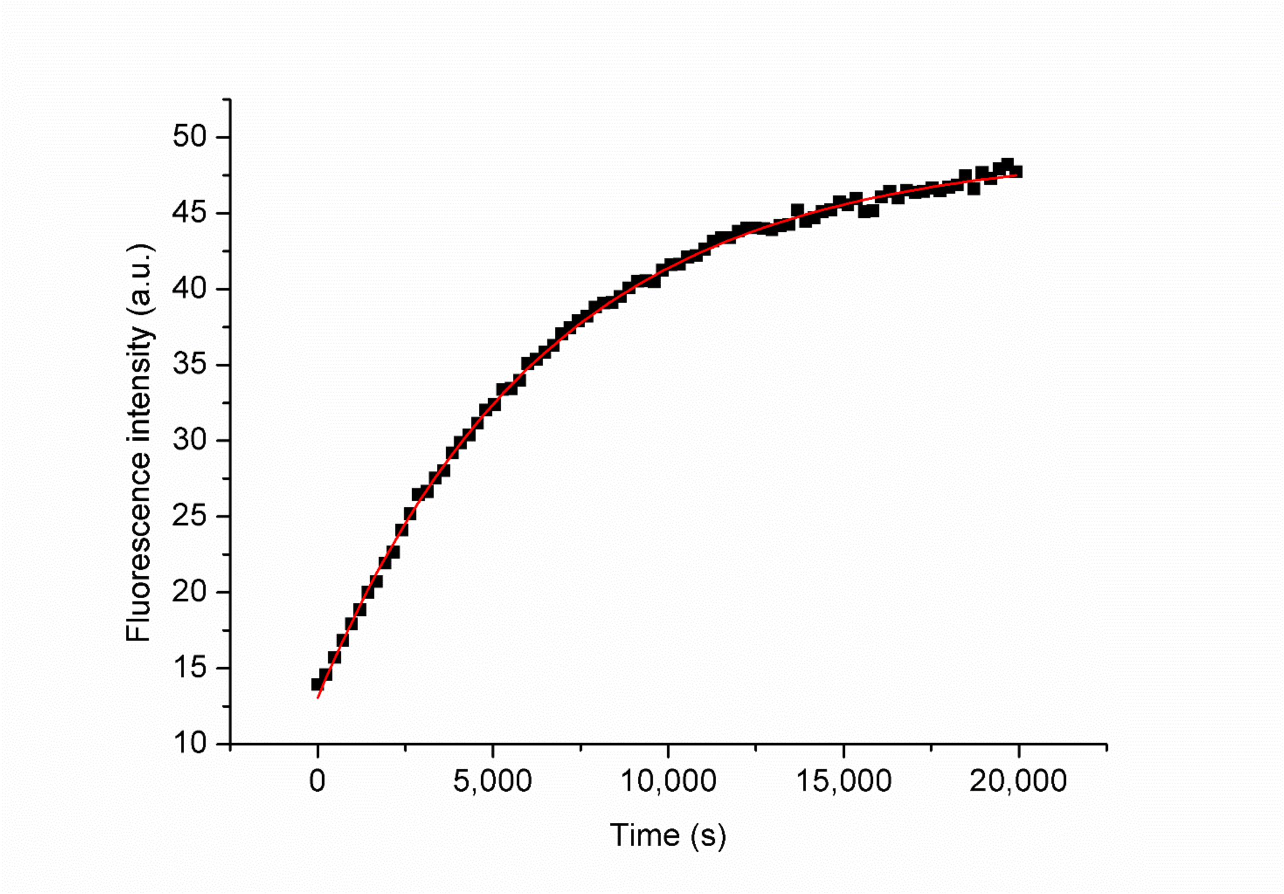

Figure S29. Representative kinetic profile of the release of CF^{3-} from POPC/TMCD 12 liposomes at 25° C.

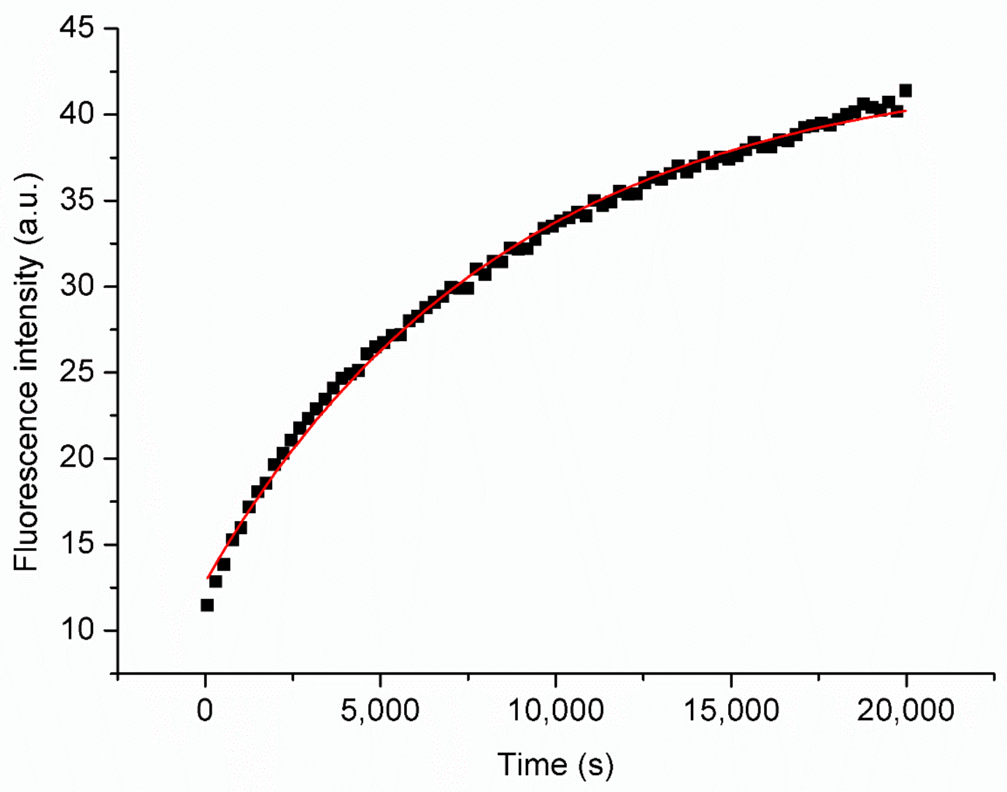

Figure S30. Representative kinetic profile of the release of CF^{3-} from POPC/TMCD 12 liposomes at 37° C.

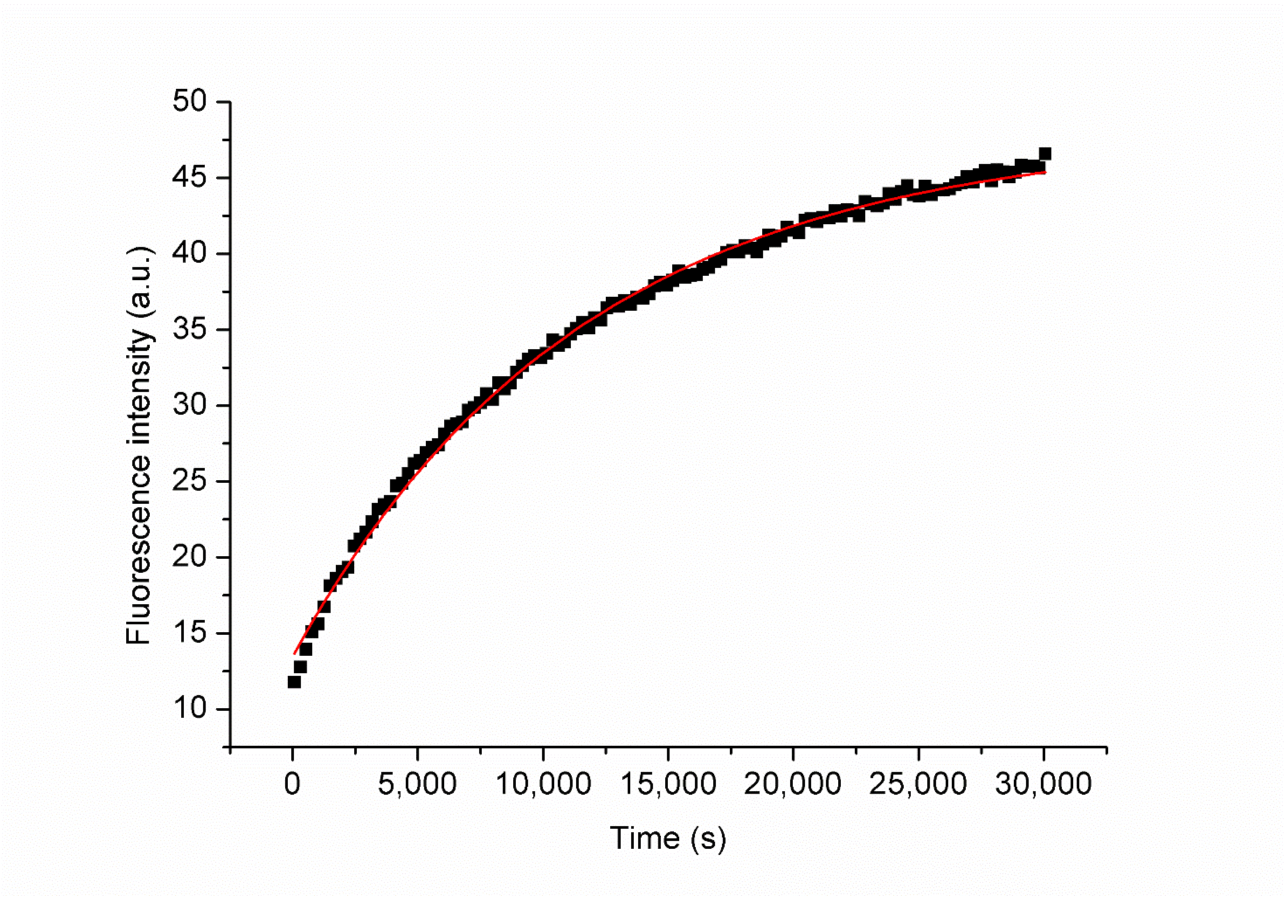

Figure S31. Representative kinetic profile of the release of CF^{3-} from POPC/TMCD 5 liposomes at 25° C.

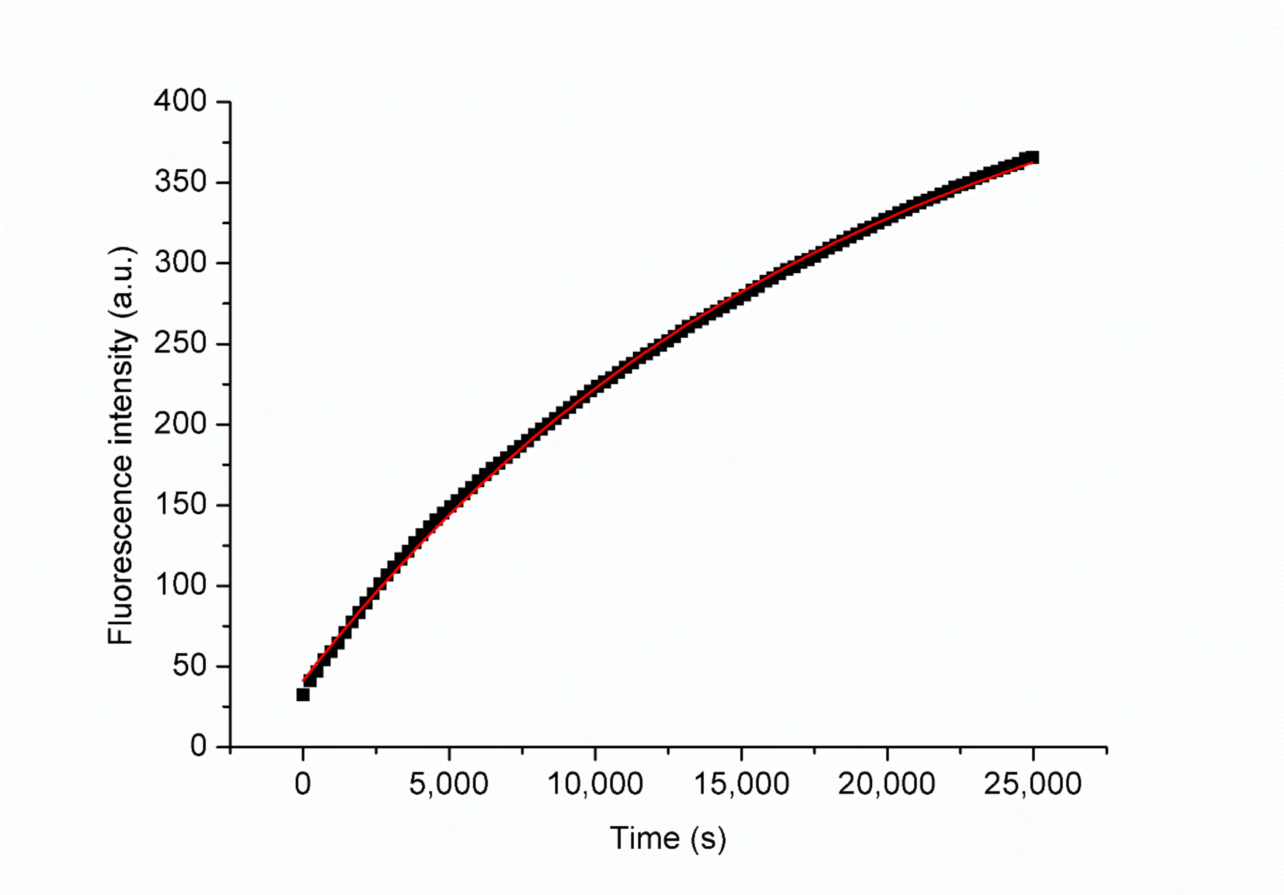

Figure S32. Representative kinetic profile of the release of CF^{3-} from POPC/TMCD 5 liposomes at 37° C.

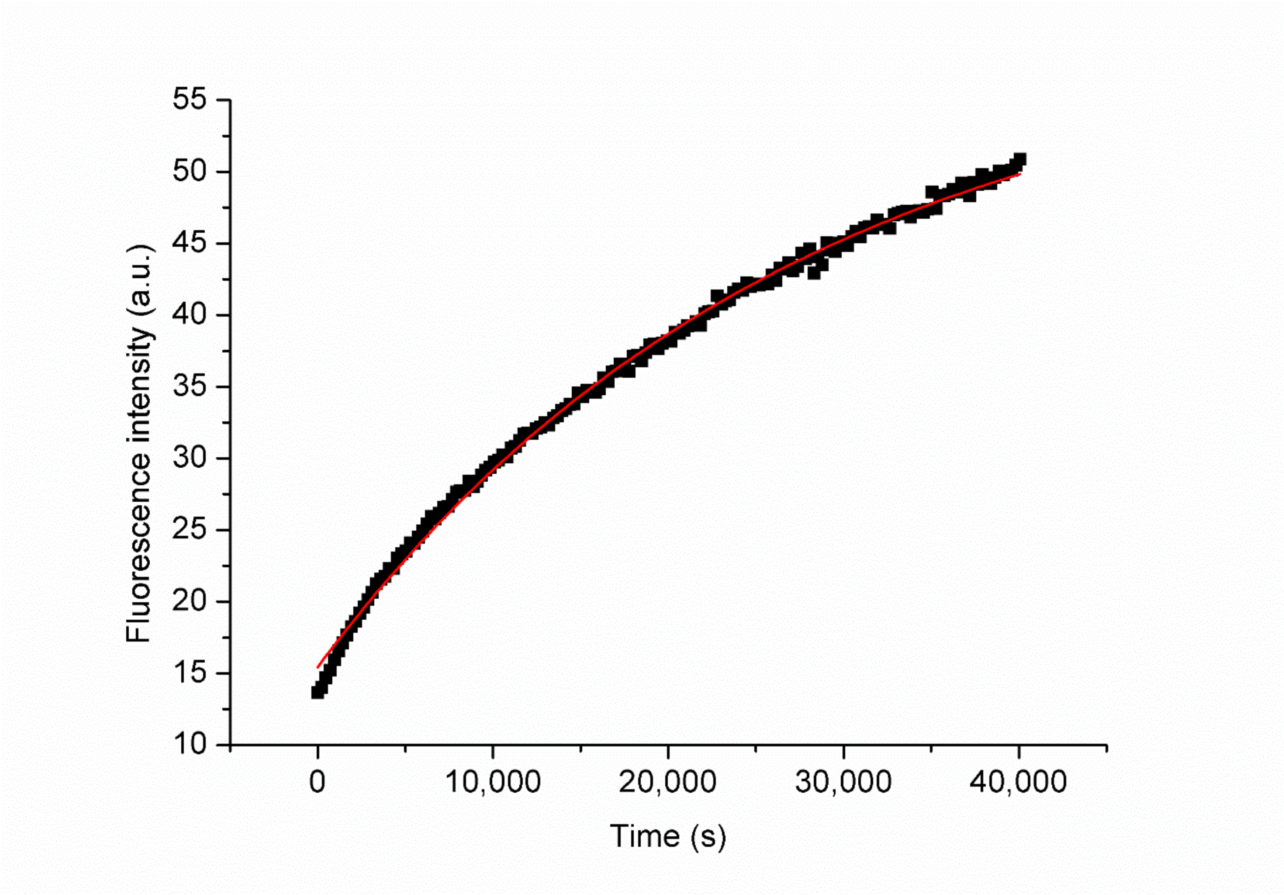

Figure S33. Representative kinetic profile of the release of CF^{3-} from POPC/TMCD 2.5 liposomes at 25° C.

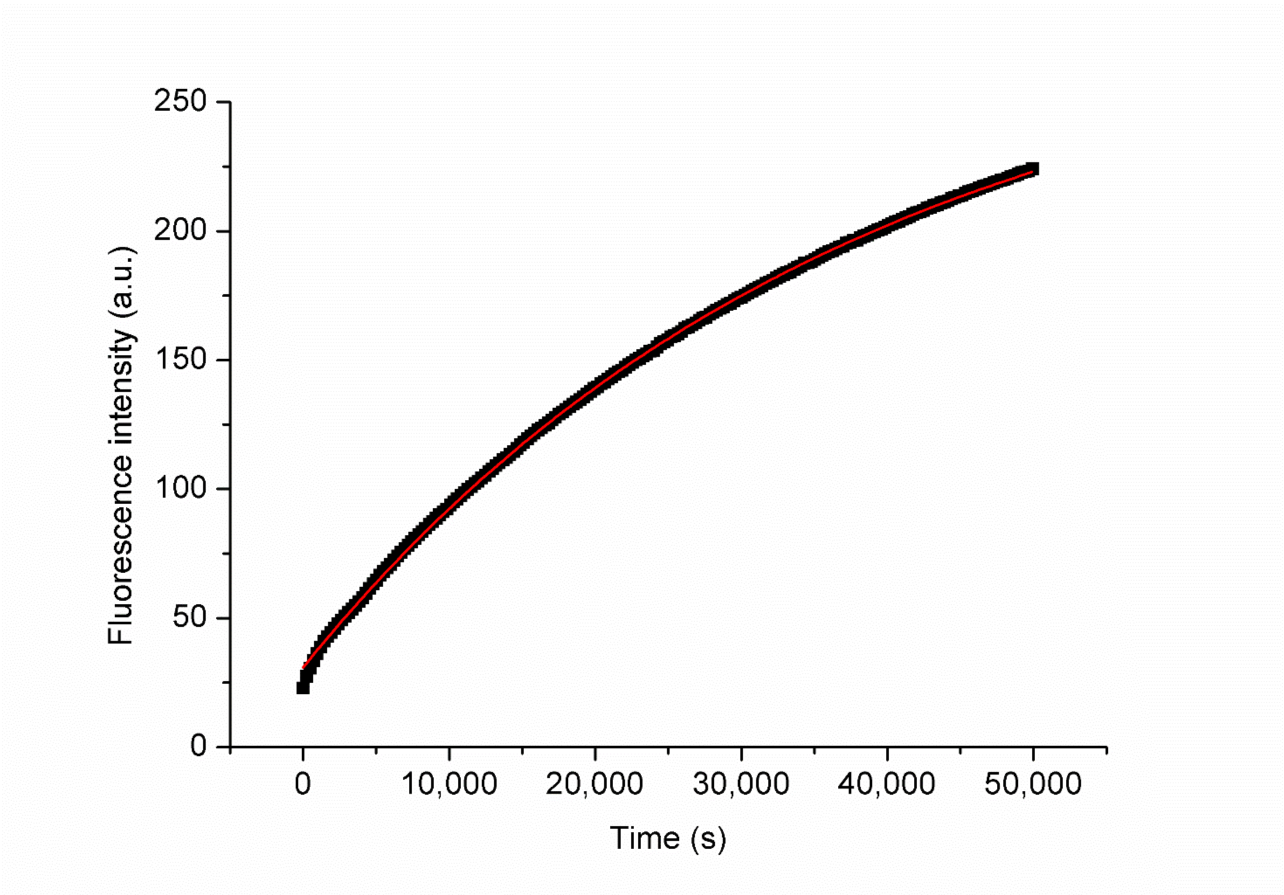

Figure S34. Representative kinetic profile of the release of CF^{3-} from POPC/TMCD 2.5 liposomes at 37° C.

S2.4 Stability measurements for POPC/DACD liposomes

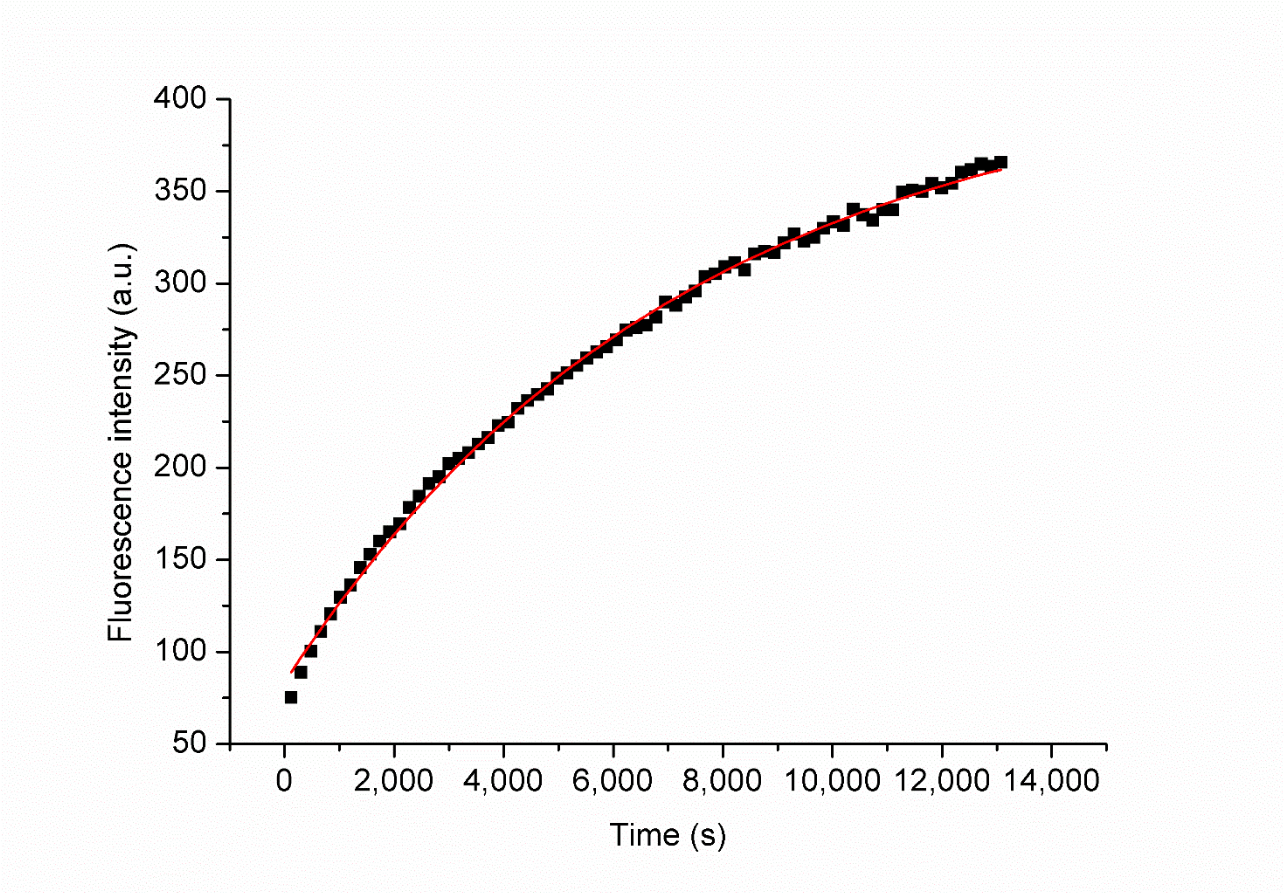

Figure S35. Representative kinetic profile of the release of CF^{3-} from POPC/DACD 12 liposomes at 25° C.

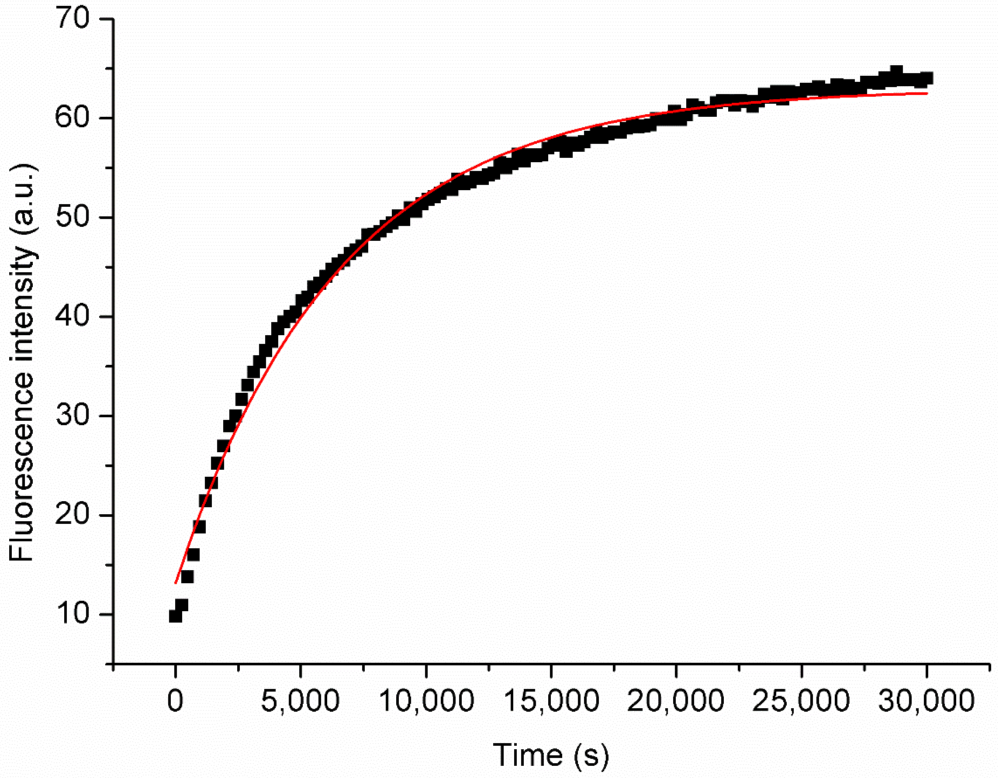

Figure S36. Representative kinetic profile of the release of CF^{3-} from POPC/DACD 12 liposomes at 37° C.

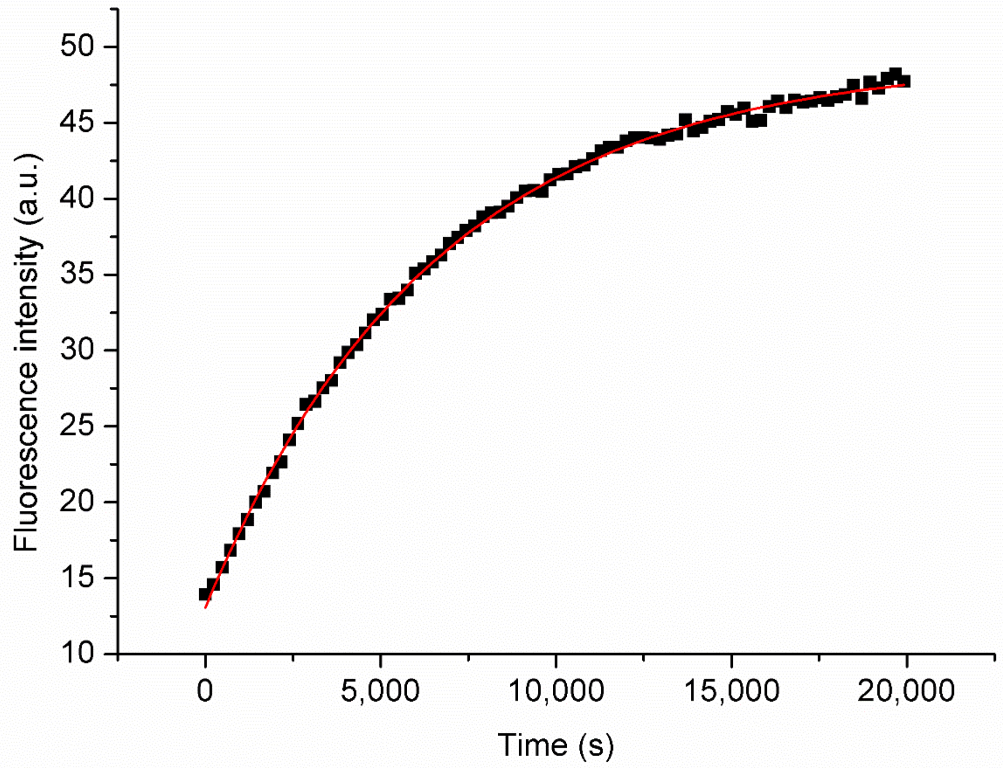

Figure S37. Representative kinetic profile of the release of CF^{3-} from POPC/DACD 5 liposomes at 25° C.

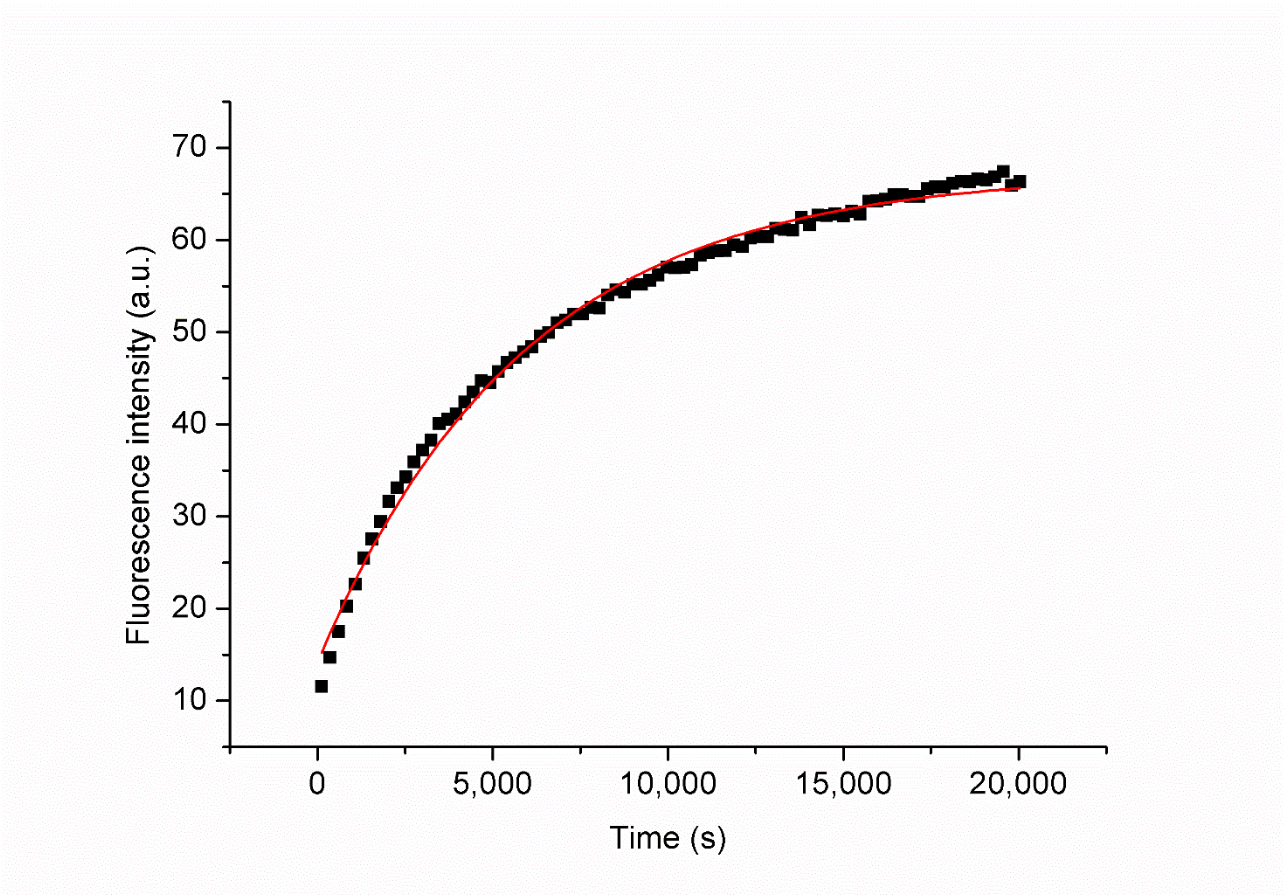

Figure S38. Representative kinetic profile of the release of CF^{3-} from POPC/DACD 5 liposomes at 37° C.

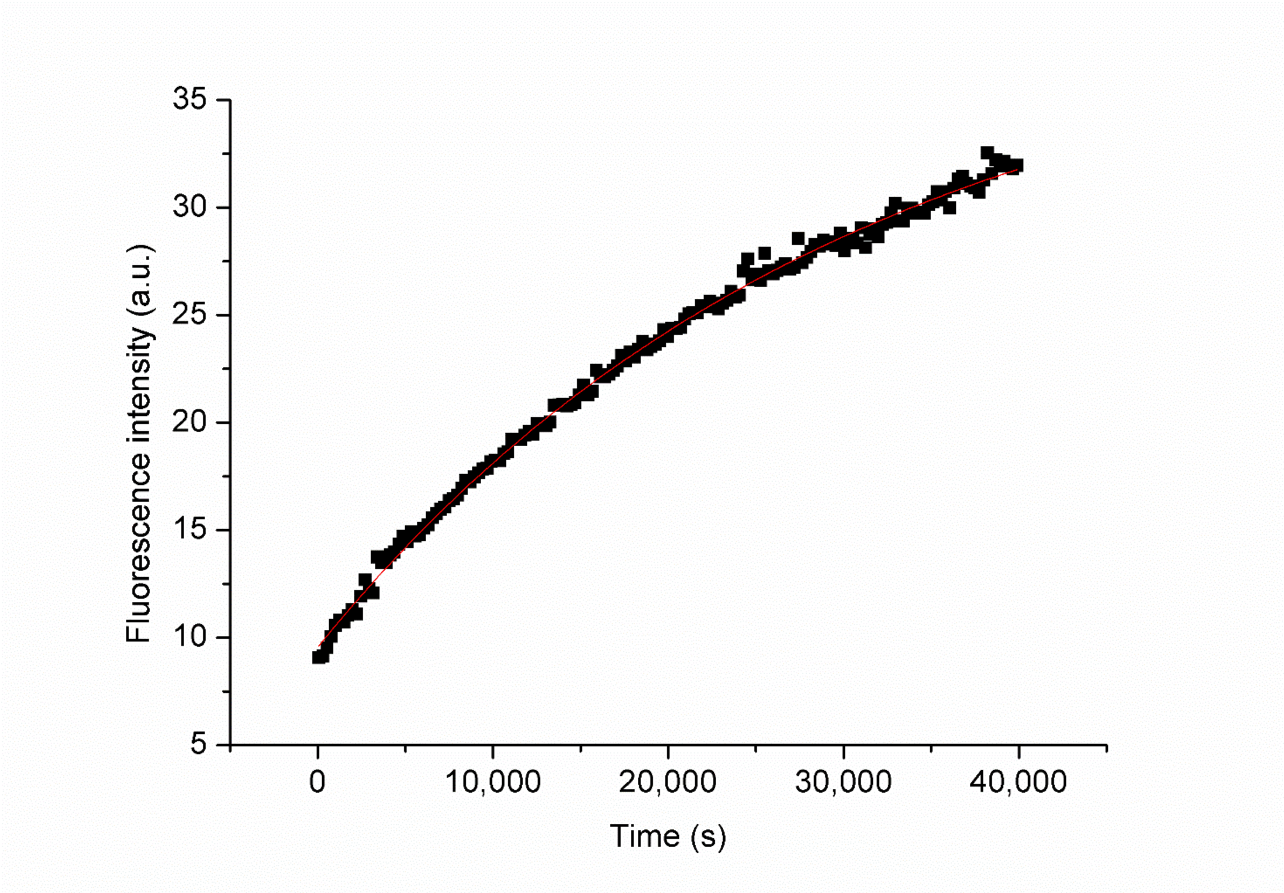

Figure S39. Representative kinetic profile of the release of CF^{3-} from POPC/DACD 2.5 liposomes at 25° C.

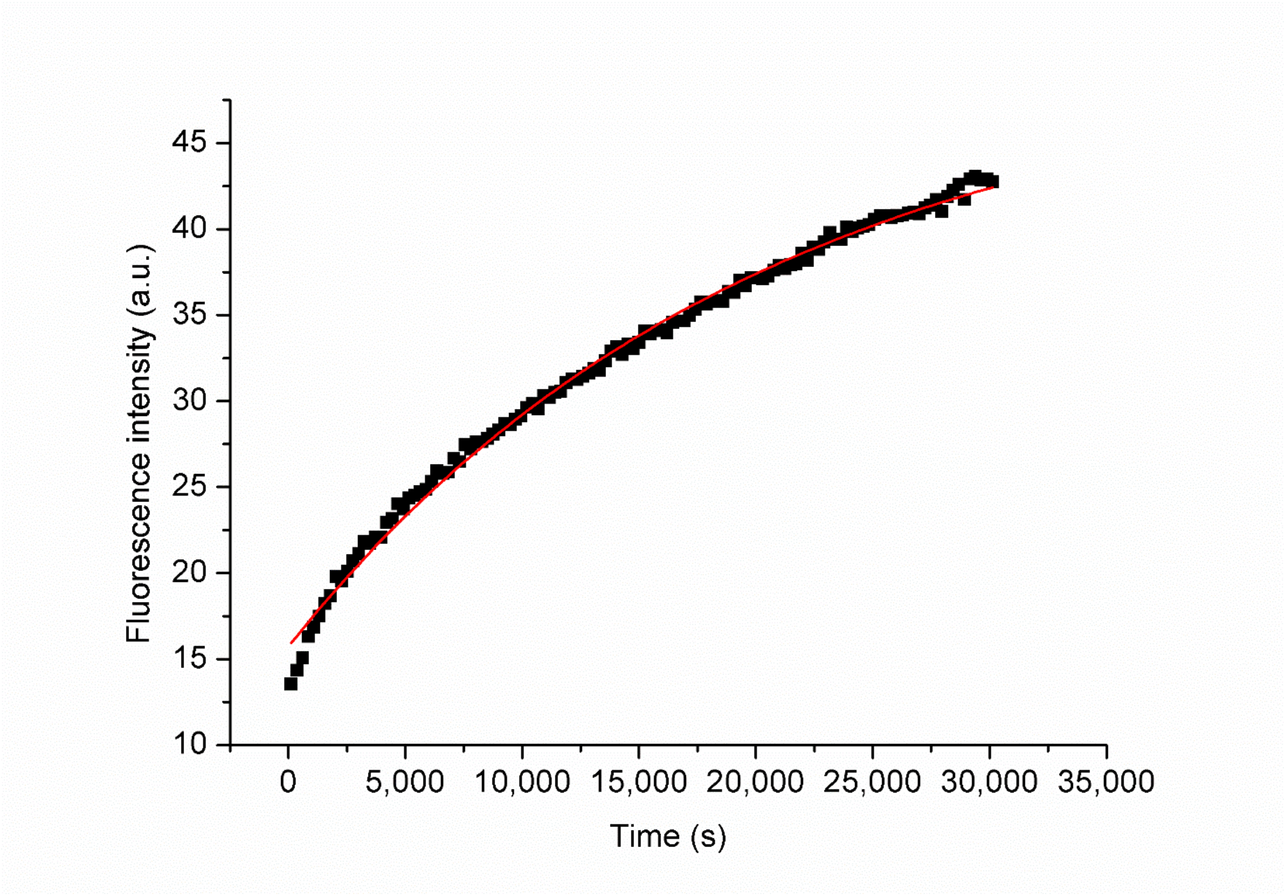

Figure S40. Representative kinetic profile of the release of CF^{3-} from POPC/DACD 2.5 liposomes at 37° C.

S3. Details of Molecular Dynamics Simulations

S3.1 Cartesian coordinates of β-CD (Angstrom).

O	23.520000	21.980000	47.910000
H	24.450000	22.340000	47.970000
C	22.840000	21.890000	49.170000
C	23.680000	21.270000	50.280000
O	24.850000	22.080000	50.480000
H	25.390000	21.680000	51.220000
C	23.020000	21.080000	51.630000
O	23.950000	20.330000	52.440000
C	21.720000	20.360000	51.310000
C	20.740000	20.190000	52.470000
O	20.840000	18.930000	53.140000
H	20.230000	18.910000	53.930000
O	20.860000	20.910000	50.290000
C	21.630000	20.970000	49.070000
O	21.970000	19.610000	48.740000
C	21.320000	19.100000	47.560000
C	20.330000	18.000000	47.920000
O	21.090000	17.000000	48.630000
C	19.400000	17.580000	46.780000
O	18.400000	18.570000	46.530000
H	17.740000	18.120000	45.940000
C	22.440000	18.540000	46.710000
O	22.150000	18.250000	45.330000
H	22.940000	18.630000	44.840000
C	23.190000	17.410000	47.390000
O	24.080000	17.990000	48.360000
H	23.530000	18.490000	49.040000
C	22.120000	16.410000	47.810000
O	22.540000	15.280000	48.600000
C	23.130000	14.140000	47.960000
C	22.360000	12.820000	48.010000
O	23.100000	11.800000	47.310000
C	20.950000	12.810000	47.420000
O	21.000000	13.130000	46.030000
H	20.320000	12.570000	45.560000
C	24.510000	13.860000	48.530000
O	25.380000	15.000000	48.550000
H	24.740000	15.760000	48.640000
C	25.260000	12.720000	47.850000
O	26.550000	12.380000	48.380000
H	27.210000	13.020000	47.980000
C	24.380000	11.480000	47.880000
O	24.130000	11.020000	49.220000
C	24.080000	9.630000	49.600000
C	23.590000	9.460000	51.030000
O	24.560000	8.770000	51.850000

C	22.270000	8.700000	51.000000
O	21.330000	9.150000	50.010000
H	20.730000	9.810000	50.470000
C	25.340000	8.850000	49.270000
O	25.050000	7.440000	49.150000
H	25.670000	7.000000	48.500000
C	26.410000	9.080000	50.320000
O	27.250000	10.210000	50.020000
H	27.070000	10.490000	49.080000
C	25.960000	9.100000	51.770000
O	25.960000	10.340000	52.500000
C	26.940000	10.560000	53.540000
C	26.310000	11.000000	54.850000
O	27.330000	11.360000	55.800000
C	25.250000	10.070000	55.440000
O	24.040000	10.120000	54.680000
H	24.290000	9.790000	53.770000
C	27.840000	11.680000	53.060000
O	28.550000	11.480000	51.820000
H	27.890000	10.980000	51.260000
C	28.890000	12.070000	54.100000
O	29.870000	12.990000	53.580000
H	29.940000	12.760000	52.610000
C	28.280000	12.390000	55.450000
O	27.460000	13.580000	55.490000
C	27.710000	14.790000	56.240000
C	26.390000	15.340000	56.760000
O	26.570000	16.660000	57.300000
C	25.720000	14.470000	57.820000
O	25.350000	13.160000	57.360000
H	26.210000	12.770000	57.050000
C	28.450000	15.810000	55.390000
O	29.780000	15.480000	54.940000
H	29.700000	14.670000	54.360000
C	28.560000	17.170000	56.050000
O	29.240000	18.140000	55.230000
H	30.020000	17.650000	54.860000
C	27.210000	17.690000	56.520000
O	26.360000	17.850000	55.360000
C	25.760000	19.140000	55.150000
C	26.400000	19.790000	53.940000
O	27.810000	19.990000	54.130000
H	28.220000	19.250000	54.660000
C	25.860000	21.200000	53.760000
O	26.510000	21.960000	52.720000
H	27.490000	21.930000	52.920000
C	24.350000	21.110000	53.570000
O	23.760000	20.460000	54.710000
C	24.260000	19.120000	54.900000
C	23.530000	18.420000	56.050000
O	22.250000	17.900000	55.680000
H	22.340000	16.980000	55.290000

S3.2 Cartesian coordinates of TMCD (Angstrom).

O	29.310000	29.210000	3.770000
C	29.210000	29.570000	2.420000
C	28.330000	29.920000	4.560000
C	28.800000	30.160000	5.980000
O	29.930000	31.050000	5.940000
C	31.140000	30.360000	6.140000
C	27.770000	30.760000	6.920000
O	28.210000	30.390000	8.240000
C	26.400000	30.160000	6.700000
C	25.300000	31.030000	7.330000
O	24.030000	30.380000	7.300000
C	23.820000	29.680000	8.490000
O	26.070000	30.010000	5.300000
C	26.990000	29.200000	4.550000
O	27.010000	27.860000	5.080000
C	26.350000	26.820000	4.330000
C	25.070000	26.330000	5.000000
O	24.500000	25.170000	4.380000
C	23.950000	27.370000	5.110000
O	23.590000	27.940000	3.840000
C	22.570000	28.880000	4.060000
C	27.300000	25.640000	4.160000
O	28.360000	26.040000	3.270000
C	29.610000	25.980000	3.900000
C	26.620000	24.420000	3.560000
O	27.480000	23.300000	3.280000
C	28.270000	22.770000	4.310000
C	25.370000	24.020000	4.330000
O	25.580000	23.500000	5.660000
C	24.670000	22.500000	6.160000
C	24.310000	22.820000	7.600000
O	23.720000	21.740000	8.340000
C	23.410000	24.050000	7.690000
O	22.210000	23.860000	6.930000
C	21.470000	25.050000	6.910000
C	25.270000	21.100000	6.040000
O	25.350000	20.660000	4.670000
C	24.150000	20.360000	4.020000
C	24.590000	20.060000	6.900000
O	25.100000	18.720000	6.790000
C	26.490000	18.520000	6.800000
C	24.480000	20.520000	8.350000
O	25.750000	20.770000	8.980000
C	26.100000	20.230000	10.270000
C	25.490000	20.980000	11.440000
O	26.450000	21.350000	12.450000
C	24.370000	20.150000	12.070000

O	23.710000	20.850000	13.120000
C	22.560000	21.470000	12.630000
C	27.620000	20.240000	10.340000
O	28.130000	19.260000	11.270000
C	28.460000	18.080000	10.590000
C	28.160000	21.620000	10.670000
O	27.960000	22.550000	9.590000
C	28.990000	22.490000	8.640000
C	27.580000	22.120000	11.980000
O	27.250000	23.520000	12.050000
C	27.780000	24.190000	13.210000
C	26.730000	25.010000	13.940000
O	27.300000	25.600000	15.120000
C	25.510000	24.190000	14.360000
O	24.490000	25.020000	14.900000
C	23.490000	24.220000	15.470000
C	28.900000	25.150000	12.840000
O	29.950000	24.500000	12.090000
C	30.170000	25.200000	10.900000
C	29.500000	25.780000	14.090000
O	30.530000	26.720000	13.750000
C	31.740000	26.380000	14.380000
C	28.420000	26.480000	14.890000
O	27.950000	27.710000	14.310000
C	27.610000	28.710000	15.290000
C	26.610000	29.690000	14.700000
O	27.020000	31.070000	14.720000
C	25.240000	29.500000	15.360000
O	25.270000	29.740000	16.770000
C	24.050000	29.350000	17.330000
C	28.810000	29.430000	15.870000
O	28.530000	29.960000	17.180000
C	29.150000	29.170000	18.150000
C	29.390000	30.520000	14.980000
O	30.430000	29.960000	14.160000
C	31.690000	30.230000	14.710000
C	28.350000	31.300000	14.200000
O	28.330000	31.030000	12.780000
C	28.630000	32.200000	12.000000
C	29.890000	31.970000	11.190000
O	30.500000	33.180000	10.720000
C	31.600000	33.520000	11.520000
C	29.730000	30.990000	10.040000
O	29.610000	29.630000	10.510000
C	30.870000	29.020000	10.540000
C	28.540000	31.410000	9.200000
O	27.360000	31.570000	10.010000
C	27.450000	32.520000	11.090000
C	27.430000	33.980000	10.640000
O	26.250000	34.300000	9.910000
C	26.220000	35.670000	9.640000

S3.3 Cartesian coordinates of DACD (Angstrom).

O	39.010000	39.650000	13.940000
C	37.680000	40.090000	13.930000
C	39.600000	39.640000	15.260000
C	41.040000	40.120000	15.370000
O	41.200000	41.350000	14.640000
C	42.010000	41.110000	13.530000
C	41.520000	40.330000	16.800000
O	42.940000	40.570000	16.690000
C	41.360000	39.000000	17.530000
C	41.890000	38.950000	18.960000
O	41.910000	37.690000	19.650000
H	40.980000	37.330000	19.720000
O	40.050000	38.430000	17.330000
C	39.750000	38.290000	15.930000
O	40.750000	37.490000	15.270000
C	40.500000	36.100000	14.990000
C	40.630000	35.090000	16.120000
O	41.990000	34.820000	16.520000
C	39.850000	33.780000	15.980000
O	39.960000	32.950000	17.130000
H	39.170000	32.320000	17.110000
C	41.330000	35.550000	13.840000
O	40.730000	34.440000	13.130000
C	40.650000	34.760000	11.770000
C	42.780000	35.260000	14.200000
O	43.510000	36.450000	14.570000
C	44.840000	36.500000	14.150000
C	42.660000	34.290000	15.360000
O	43.950000	33.810000	15.760000
C	44.110000	32.690000	16.640000
C	45.220000	33.000000	17.640000
O	45.730000	31.900000	18.410000
C	44.850000	34.020000	18.720000
O	44.390000	35.300000	18.290000
H	43.480000	35.250000	17.880000
C	44.450000	31.370000	15.980000
O	43.470000	31.000000	14.990000
C	43.900000	30.970000	13.660000
C	44.860000	30.230000	16.900000
O	45.010000	28.920000	16.320000
C	43.910000	28.060000	16.230000
C	46.080000	30.740000	17.630000
O	47.290000	31.050000	16.900000
C	48.530000	30.620000	17.490000
C	48.970000	31.480000	18.670000
O	49.220000	32.830000	18.250000
C	50.130000	30.910000	19.500000
O	50.760000	31.770000	20.460000
H	50.290000	32.050000	21.290000

C	49.470000	30.720000	16.310000
O	50.750000	30.050000	16.300000
C	50.640000	28.740000	15.810000
C	49.650000	32.180000	15.910000
O	48.550000	32.860000	15.270000
C	48.860000	33.490000	14.060000
C	50.080000	33.000000	17.120000
O	50.220000	34.420000	16.890000
C	50.900000	35.190000	17.890000
C	50.090000	36.370000	18.420000
O	50.830000	37.100000	19.420000
C	48.700000	36.000000	18.930000
O	48.720000	34.920000	19.870000
H	48.940000	34.150000	19.270000
C	52.180000	35.820000	17.370000
O	53.070000	34.810000	16.860000
C	53.580000	35.150000	15.610000
C	52.890000	36.650000	18.420000
O	54.100000	37.260000	17.940000
C	55.250000	36.650000	18.460000
C	51.910000	37.770000	18.760000
O	51.490000	38.750000	17.790000
C	51.510000	40.110000	18.250000
C	50.460000	40.460000	19.300000
O	49.090000	40.440000	18.850000
C	50.910000	41.660000	20.130000
O	51.970000	41.260000	21.010000
H	51.650000	40.810000	21.850000
C	51.220000	40.840000	16.950000
O	51.460000	42.250000	17.180000
C	52.250000	42.800000	16.170000
C	49.820000	40.530000	16.450000
O	49.590000	39.250000	15.840000
C	50.090000	39.280000	14.540000
C	48.830000	40.940000	17.520000
O	47.500000	40.410000	17.330000
C	46.400000	41.290000	17.610000
C	45.760000	41.880000	16.370000
O	46.640000	42.690000	15.570000
C	46.630000	42.230000	14.240000
C	44.470000	42.570000	16.770000
O	43.840000	43.410000	15.780000
C	43.550000	44.670000	16.330000
C	43.460000	41.670000	17.460000
O	44.120000	41.160000	18.630000
C	45.380000	40.500000	18.420000
C	45.920000	40.070000	19.780000
O	46.980000	39.130000	19.690000
H	47.720000	39.500000	19.120000
O	36.880000	39.210000	14.040000
C	37.190000	41.400000	13.780000
O	42.210000	40.090000	12.930000

C	42.330000	42.290000	12.850000
O	40.730000	35.880000	11.350000
C	40.380000	33.760000	10.830000
O	45.170000	36.100000	13.070000
C	45.760000	37.160000	14.970000
O	44.920000	30.410000	13.380000
C	42.890000	31.320000	12.750000
O	42.920000	28.130000	16.920000
C	44.100000	26.960000	15.390000
O	49.750000	28.440000	15.070000
C	51.620000	27.810000	16.160000
O	49.520000	32.890000	13.260000
C	48.460000	34.800000	13.770000
O	53.320000	36.190000	15.070000
C	54.210000	34.150000	14.850000
O	55.270000	36.440000	19.640000
C	56.370000	36.710000	17.630000
O	52.220000	42.310000	15.070000
C	52.860000	44.050000	16.370000
O	50.030000	40.260000	13.840000
C	50.520000	38.130000	13.870000
O	45.600000	41.900000	13.730000
C	47.740000	42.390000	13.400000
O	44.380000	45.320000	16.910000
C	42.310000	45.310000	16.380000

S3.4 Atom-types and charges for topology of β-CD (see S3.1).

```
 [ atoms ]
; nr   type  resnr resid  atom  cgnr  charge    mass
   1    OA    1  2   OBX    1  -0.60  15.9994
   2     H    1  2   HBX    1   0.42   1.0080
   3   CH1    1  2   CBS    1   0.1 13.0190
   4   CH1    1  2   CBR    1   0.45 13.0190
   5    OA    1  2   OBY    1  -0.60 15.9994
   6     H    1  2   HBY    1   0.43  1.0080
   7   CH1    1  2   CBQ    1   0.10 13.0190
   8    OA    1  2   OBP    2  -0.64 15.9994
   9   CH1    1  2   CBV    2   0.38 13.0190
  10   CH2    1  2   CBZ    2   0.16 14.0270
  11    OA    1  2   OCA    2  -0.64 15.9994
```

12	H	1	2	HCA	2	0.43	1.0080
13	OA	1	2	OBU	2	-0.50	15.9994
14	CH1	1	2	CBT	2	0.45	13.0190
15	OA	1	2	O4	2	-0.58	15.9994
16	CH1	1	2	C4	3	0.10	13.0190
17	CH1	1	2	C5	3	0.38	13.0190
18	OA	1	2	O5	3	-0.50	15.9994
19	CH2	1	2	C6	3	0.16	14.0270
20	OA	1	2	O6	3	-0.64	15.9994
21	H	1	2	H63	3	0.43	1.0080
22	CH1	1	2	C3	3	0.45	13.0190
23	OA	1	2	O3	3	-0.60	15.9994
24	H	1	2	H32	3	0.43	1.0080
25	CH1	1	2	C2	4	0.10	13.0190
26	OA	1	2	O2	4	-0.60	15.9994
27	H	1	2	H22	4	0.43	1.0080
28	CH1	1	2	C1	4	0.45	13.0190
29	OA	1	2	O1	4	-0.58	15.9994
30	CH1	1	2	CAH	4	0.10	13.0190
31	CH1	1	2	CAM	4	0.38	13.0190
32	OA	1	2	OAL	4	-0.50	15.9994
33	CH2	1	2	CCT	5	0.16	14.0270
34	OA	1	2	OCU	5	-0.64	15.9994
35	H	1	2	HCU	5	0.43	1.0080
36	CH1	1	2	CAI	6	0.45	13.0190
37	OA	1	2	OCS	6	-0.60	15.9994
38	H	1	2	HCS	6	0.43	1.0080
39	CH1	1	2	CAJ	7	0.10	13.0190
40	OA	1	2	OCR	7	-0.60	15.9994
41	H	1	2	HCR	7	0.43	1.0080
42	CH1	1	2	CAK	7	0.45	13.0190
43	OA	1	2	OAN	7	-0.58	15.9994
44	CH1	1	2	CAO	7	0.10	13.0190

45	CH1	1	2	CAT	7	0.38	13.0190
46	OA	1	2	OAS	7	-0.50	15.9994
47	CH2	1	2	CCP	8	0.16	14.0270
48	OA	1	2	OCQ	8	-0.64	15.9994
49	H	1	2	HCQ	8	0.43	1.0080
50	CH1	1	2	CAP	9	0.45	13.0190
51	OA	1	2	OCO	9	-0.60	15.9994
52	H	1	2	HCO	9	0.43	1.0080
53	CH1	1	2	CAQ	10	0.10	13.0190
54	OA	1	2	OCN	10	-0.60	15.9994
55	H	1	2	HCN	10	0.43	1.0080
56	CH1	1	2	CAR	10	0.45	13.0190
57	OA	1	2	OAU	10	-0.58	15.9994
58	CH1	1	2	CAV	10	0.10	13.0190
59	CH1	1	2	CBA	10	0.38	13.0190
60	OA	1	2	OAZ	10	-0.50	15.9994
61	CH2	1	2	CCL	11	0.16	14.0270
62	OA	1	2	OCM	11	-0.64	15.9994
63	H	1	2	HCM	11	0.43	1.0080
64	CH1	1	2	CAW	12	0.45	13.0190
65	OA	1	2	OCK	12	-0.60	15.9994
66	H	1	2	HCK	12	0.43	1.0080
67	CH1	1	2	CAX	13	0.10	13.0190
68	OA	1	2	OCJ	13	-0.60	15.9994
69	H	1	2	HCJ	13	0.43	1.0080
70	CH1	1	2	CAY	13	0.45	13.0190
71	OA	1	2	OBB	13	-0.58	15.9994
72	CH1	1	2	CBC	13	0.10	13.0190
73	CH1	1	2	CBH	13	0.38	13.0190
74	OA	1	2	OBG	13	-0.50	15.9994
75	CH2	1	2	CCH	14	0.16	14.0270
76	OA	1	2	OCI	14	-0.64	15.9994
77	H	1	2	HCI	14	0.43	1.0080

```
78    CH1   1  2   CBD   14   0.45 13.0190
79    OA    1  2   OCG   14   -0.60 15.9994
80    H     1  2   HCG   14   0.43  1.0080
81    CH1   1  2   CBE   14   0.10 13.0190
82    OA    1  2   OCF   14   -0.60 15.9994
83    H     1  2   HCF   14   0.43  1.0080
84    CH1   1  2   CBF   14   0.45 13.0190
85    OA    1  2   OBI   15   -0.58 15.9994
86    CH1   1  2   CBJ   15   0.10 13.0190
87    CH1   1  2   CBK   15   0.45 13.0190
88    OA    1  2   OCC   15   -0.60 15.9994
89    H     1  2   HCC   15   0.43 1.0080
90    CH1   1  2   CBL   16   0.10 13.0190
91    OA    1  2   OCB   16   -0.60 15.9994
92    H     1  2   HCB   16   0.43 1.0080
93    CH1   1  2   CBM   16   0.45 13.0190
94    OA    1  2   OBN   16   -0.50 15.9994
95    CH1   1  2   CBO   16   0.38 13.0190
96    CH2   1  2   CCD   16   0.16 14.0270
97    OA    1  2   OCE   16   -0.64 15.9994
98    H     1  2   HCE   16   0.43 1.0080
```

S3.5 Atom-types and charges for topology of TMCD (see S3.1).

```
 [ atoms ]
;  nr   type  resnr resid  atom  cgnr   charge    mass
   1    OA    1  _2   OBX   1  -0.18  15.9994
   2    CH3   1  _2    C    1   0.05  15.000
   3    CH1   1  _2   CBS   1   0.1 13.0190
   4    CH1   1  _2   CBR   1   0.30 13.0190
   5    OA    1  _2   OBY   1   -0.20 15.9994
   6    CH3   1  _2   HBX   1   0.05  15.000
   7    CH1   1  _2   CBQ   1   0.10 13.0190
```

8	OA	1	_2	OBP	2	-0.20	15.9994
9	CH1	1	_2	CBV	2	0.22	13.0190
10	CH2	1	_2	CBZ	2	0.16	14.0270
11	OA	1	_2	OCA	2	-0.20	15.9994
12	CH3	1	_2	HBX	2	0.05	15.000
13	OA	1	_2	OBU	2	-0.40	15.9994
14	CH1	1	_2	CBT	2	0.30	13.0190
15	OA	1	_2	O4	2	-0.35	15.9994
16	CH1	1	_2	C4	3	0.10	13.0190
17	CH1	1	_2	C5	3	0.22	13.0190
18	OA	1	_2	O5	3	-0.40	15.9994
19	CH2	1	_2	C6	3	0.16	14.0270
20	OA	1	_2	O6	3	-0.20	15.9994
21	CH3	1	_2	HBX	3	0.05	15.000
22	CH1	1	_2	C3	3	0.30	13.0190
23	OA	1	_2	O3	3	-0.20	15.9994
24	CH3	1	_2	HBX	3	0.05	15.000
25	CH1	1	_2	C2	4	0.10	13.0190
26	OA	1	_2	O2	4	-0.20	15.9994
27	CH3	1	_2	HBX	4	0.05	15.000
28	CH1	1	_2	C1	4	0.30	13.0190
29	OA	1	_2	O1	4	-0.35	15.9994
30	CH1	1	_2	CAH	4	0.10	13.0190
31	CH1	1	_2	CAM	4	0.22	13.0190
32	OA	1	_2	OAL	4	-0.40	15.9994
33	CH2	1	_2	CCT	5	0.16	14.0270
34	OA	1	_2	OCU	5	-0.20	15.9994
35	CH3	1	_2	HBX	5	0.05	15.000
36	CH1	1	_2	CAI	6	0.30	13.0190
37	OA	1	_2	OCS	6	-0.20	15.9994
38	CH3	1	_2	HBX	6	0.05	15.000
39	CH1	1	_2	CAJ	7	0.10	13.0190
40	OA	1	_2	OCR	7	-0.20	15.9994

41	CH3	1	_2	HBX	7	0.05	15.000
42	CH1	1	_2	CAK	7	0.30	13.0190
43	OA	1	_2	OAN	7	-0.35	15.9994
44	CH1	1	_2	CAO	7	0.10	13.0190
45	CH1	1	_2	CAT	7	0.22	13.0190
46	OA	1	_2	OAS	7	-0.40	15.9994
47	CH2	1	_2	CCP	8	0.16	14.0270
48	OA	1	_2	OCQ	8	-0.20	15.9994
49	CH3	1	_2	HBX	8	0.05	15.000
50	CH1	1	_2	CAP	9	0.30	13.0190
51	OA	1	_2	OCO	9	-0.20	15.9994
52	CH3	1	_2	HBX	9	0.05	15.000
53	CH1	1	_2	CAQ	10	0.10	13.0190
54	OA	1	_2	OCN	10	-0.20	15.9994
55	CH3	1	_2	HBX	10	0.05	15.000
56	CH1	1	_2	CAR	10	0.30	13.0190
57	OA	1	_2	OAU	10	-0.35	15.9994
58	CH1	1	_2	CAV	10	0.10	13.0190
59	CH1	1	_2	CBA	10	0.22	13.0190
60	OA	1	_2	OAZ	10	-0.40	15.9994
61	CH2	1	_2	CCL	11	0.16	14.0270
62	OA	1	_2	OCM	11	-0.20	15.9994
63	CH3	1	_2	HBX	11	0.05	15.000
64	CH1	1	_2	CAW	12	0.30	13.0190
65	OA	1	_2	OCK	12	-0.20	15.9994
66	CH3	1	_2	HBX	12	0.05	15.000
67	CH1	1	_2	CAX	13	0.10	13.0190
68	OA	1	_2	OCJ	13	-0.20	15.9994
69	CH3	1	_2	HBX	13	0.04	15.000
70	CH1	1	_2	CAY	13	0.30	13.0190
71	OA	1	_2	OBB	13	-0.35	15.9994
72	CH1	1	_2	CBC	13	0.10	13.0190
73	CH1	1	_2	CBH	13	0.22	13.0190

74	OA	1	_2	OBG	13	-0.40	15.9994
75	CH2	1	_2	CCH	14	0.16	14.0270
76	OA	1	_2	OCI	14	-0.20	15.9994
77	CH3	1	_2	HBX	14	0.05	15.000
78	CH1	1	_2	CBD	14	0.30	13.0190
79	OA	1	_2	OCG	14	-0.20	15.9994
80	CH3	1	_2	HBX	14	0.04	15.000
81	CH1	1	_2	CBE	14	0.10	13.0190
82	OA	1	_2	OCF	14	-0.20	15.9994
83	CH3	1	_2	HBX	14	0.05	15.000
84	CH1	1	_2	CBF	14	0.30	13.0190
85	OA	1	_2	OBI	15	-0.35	15.9994
86	CH1	1	_2	CBJ	15	0.10	13.0190
87	CH1	1	_2	CBK	15	0.30	13.0190
88	OA	1	_2	OCC	15	-0.20	15.9994
89	CH3	1	_2	HBX	15	0.05	15.000
90	CH1	1	_2	CBL	16	0.10	13.0190
91	OA	1	_2	OCB	16	-0.20	15.9994
92	CH3	1	_2	HBX	16	0.05	15.000
93	CH1	1	_2	CBM	16	0.30	13.0190
94	OA	1	_2	OBN	16	-0.40	15.9994
95	CH1	1	_2	CBO	16	0.22	13.0190
96	CH2	1	_2	CCD	16	0.16	14.0270
97	OA	1	_2	OCE	16	-0.20	15.9994
98	CH3	1	_2	HBX	16	0.04	15.000

S3.6 Atom-types and charges for topology of DACD (see S3.1).

[atoms]

; nr	type	resnr	resid	atom	cgnr	charge	mass
1	OA	1	_2	OBX	1	-0.50	15.9994
2	C	1	_2	C	1	0.70	12.0080
3	CH1	1	_2	CBS	1	0.1074	13.0190

4	CH1	1	_2	CBR	1	0.45	13.0190
5	OA	1	_2	OBY	1	-0.50	15.9994
6	C	1	_2	C	1	0.70	12.0080
7	CH1	1	_2	CBQ	1	0.10	13.0190
8	OA	1	_2	OBP	2	-0.50	15.9994
9	CH1	1	_2	CBV	2	0.38	13.0190
10	CH2	1	_2	CBZ	2	0.16	14.0270
11	OA	1	_2	OCA	2	-0.50	15.9994
12	H	1	_2	HCA	2	0.43	1.0080
13	OA	1	_2	OBU	2	-0.50	15.9994
14	CH1	1	_2	CBT	2	0.45	13.0190
15	OA	1	_2	O4	2	-0.55	15.9994
16	CH1	1	_2	C4	3	0.10	13.0190
17	CH1	1	_2	C5	3	0.38	13.0190
18	OA	1	_2	O5	3	-0.50	15.9994
19	CH2	1	_2	C6	3	0.16	14.0270
20	OA	1	_2	O6	3	-0.50	15.9994
21	H	1	_2	H63	3	0.43	1.0080
22	CH1	1	_2	C3	3	0.45	13.0190
23	OA	1	_2	O3	3	-0.50	15.9994
24	C	1	_2	C	3	0.70	12.0080
25	CH1	1	_2	C2	4	0.10	13.0190
26	OA	1	_2	O2	4	-0.50	15.9994
27	C	1	_2	C	4	0.70	12.080
28	CH1	1	_2	C1	4	0.45	13.0190
29	OA	1	_2	O1	4	-0.55	15.9994
30	CH1	1	_2	CAH	4	0.10	13.0190
31	CH1	1	_2	CAM	4	0.38	13.0190
32	OA	1	_2	OAL	4	-0.50	15.9994
33	CH2	1	_2	CCT	5	0.16	14.0270
34	OA	1	_2	OCU	5	-0.50	15.9994
35	H	1	_2	HCU	5	0.43	1.0080
36	CH1	1	_2	CAI	6	0.45	13.0190

37	OA	1	_2	OCS	6	-0.50	15.9994
38	C	1	_2	C	6	0.70	12.080
39	CH1	1	_2	CAJ	7	0.10	13.0190
40	OA	1	_2	OCR	7	-0.50	15.9994
41	C	1	_2	C	7	0.70	12.080
42	CH1	1	_2	CAK	7	0.45	13.0190
43	OA	1	_2	OAN	7	-0.55	15.9994
44	CH1	1	_2	CAO	7	0.10	13.0190
45	CH1	1	_2	CAT	7	0.38	13.0190
46	OA	1	_2	OAS	7	-0.50	15.9994
47	CH2	1	_2	CCP	8	0.16	14.0270
48	OA	1	_2	OCQ	8	-0.50	15.9994
49	H	1	_2	HCQ	8	0.43	1.0080
50	CH1	1	_2	CAP	9	0.45	13.0190
51	OA	1	_2	OCO	9	-0.50	15.9994
52	C	1	_2	C	9	0.70	12.080
53	CH1	1	_2	CAQ	10	0.10	13.0190
54	OA	1	_2	OCN	10	-0.50	15.9994
55	C	1	_2	C	10	0.70	12.080
56	CH1	1	_2	CAR	10	0.45	13.0190
57	OA	1	_2	OAU	10	-0.55	15.9994
58	CH1	1	_2	CAV	10	0.10	13.0190
59	CH1	1	_2	CBA	10	0.38	13.0190
60	OA	1	_2	OAZ	10	-0.50	15.9994
61	CH2	1	_2	CCL	11	0.16	14.0270
62	OA	1	_2	OCM	11	-0.50	15.9994
63	H	1	_2	HCM	11	0.43	1.0080
64	CH1	1	_2	CAW	12	0.45	13.0190
65	OA	1	_2	OCK	12	-0.50	15.9994
66	C	1	_2	C	12	0.70	12.080
67	CH1	1	_2	CAX	13	0.10	13.0190
68	OA	1	_2	OCJ	13	-0.50	15.9994
69	C	1	_2	C	13	0.70	12.080

70	CH1	1	_2	CAY	13	0.45	13.0190
71	OA	1	_2	OBB	13	-0.55	15.9994
72	CH1	1	_2	CBC	13	0.10	13.0190
73	CH1	1	_2	CBH	13	0.38	13.0190
74	OA	1	_2	OBG	13	-0.50	15.9994
75	CH2	1	_2	CCH	14	0.16	14.0270
76	OA	1	_2	OCI	14	-0.50	15.9994
77	H	1	_2	HCI	14	0.43	1.0080
78	CH1	1	_2	CBD	14	0.45	13.0190
79	OA	1	_2	OCG	14	-0.50	15.9994
80	C	1	_2	C	14	0.70	12.0080
81	CH1	1	_2	CBE	14	0.10	13.0190
82	OA	1	_2	OCF	14	-0.50	15.9994
83	C	1	_2	C	14	0.70	12.0080
84	CH1	1	_2	CBF	14	0.45	13.0190
85	OA	1	_2	OBI	15	-0.55	15.9994
86	CH1	1	_2	CBJ	15	0.10	13.0190
87	CH1	1	_2	CBK	15	0.45	13.0190
88	OA	1	_2	OCC	15	-0.50	15.9994
89	C	1	_2	C	15	0.70	12.080
90	CH1	1	_2	CBL	16	0.10	13.0190
91	OA	1	_2	OCB	16	-0.50	15.9994
92	C	1	_2	C	16	0.70	12.080
93	CH1	1	_2	CBM	16	0.45	13.0190
94	OA	1	_2	OBN	16	-0.50	15.9994
95	CH1	1	_2	CBO	16	0.38	13.0190
96	CH2	1	_2	CCD	16	0.16	14.0270
97	OA	1	_2	OCE	16	-0.50	15.9994
98	H	1	_2	HCE	16	0.43	1.0080
99	O	1	_2	O	17	-0.56	16.00
100	CH3	1	_2	C	18	0.0959	15.
101	O	1	_2	O	19	-0.56	16.00
102	CH3	1	_2	C	20	0.0959	15.

103	O	1	_2	O	21	-0.56	16.00
104	CH3	1	_2	C	22	0.0959	15.
105	O	1	_2	O	23	-0.56	16.00
106	CH3	1	_2	C	24	0.0959	15.
107	O	1	_2	O	25	-0.56	16.00
108	CH3	1	_2	C	26	0.0959	15.
109	O	1	_2	O	27	-0.56	16.00
110	CH3	1	_2	C	28	0.0959	15.
111	O	1	_2	O	29	-0.56	16.00
112	CH3	1	_2	C	30	0.0959	15.
113	O	1	_2	O	31	-0.56	16.00
114	CH3	1	_2	C	32	0.0959	15.
115	O	1	_2	O	33	-0.56	16.00
116	CH3	1	_2	C	34	0.0959	15.
117	O	1	_2	O	35	-0.56	16.00
118	CH3	1	_2	C	36	0.0959	15.
119	O	1	_2	O	37	-0.56	16.00
120	CH3	1	_2	C	38	0.0959	15.
121	O	1	_2	O	39	-0.56	16.00
122	CH3	1	_2	C	40	0.0959	15.
123	O	1	_2	O	41	-0.56	16.00
124	CH3	1	_2	C	42	0.0959	15.
125	O	1	_2	O	43	-0.56	16.00
126	CH3	1	_2	C	44	0.0959	15.
